# Supplementary material for: Frustrated Lewis pair catalyst realizes efficient green diesel production
Source: Nat Commun. 2024 Apr 12;15:3172. doi: 10.1038/s41467-024-47591-z (PMC11015034; doi:10.1038/s41467-024-47591-z)
Supplement: Supplementary file 1 — Supplementary Information [file 41467_2024_47591_MOESM1_ESM.pdf]

Supplementary Materials for

# Frustrated Lewis pair catalyst realizes efficient green diesel production

De-Chang Li[1,2,3,4], Zhengyi Pan[1,2,3,4], Zhengbin Tian[1,2,3], Qian Zhang[1,2,3,4], Xiaohui Deng[1,2,3], Heqing Jiang[1,2,3,4] & Guang-Hui Wang*[,1,2,3,4]

[1]Qingdao Institute of Bioenergy and Bioprocess Technology, Chinese Academy of Sciences, 266101 Qingdao, China.

[2]Shandong Energy Institute, 266101 Qingdao, China.

[3]Qingdao New Energy Shandong Laboratory, 266101 Qingdao, China.

[4]University of Chinese Academy of Sciences, 100049 Beijing, China

*Corresponding author. Email: wanggh@qibebt.ac.cn.

**Materials**

$Ni(NO_3)_2$ $6H_2O$, $Al(NO_3)_3$ $9H_2O$, urea, $NaH_2PO_2$, hydrochloric acid and $NH_4H_2PO_4$ were purchased from the Sinopharm Chemical Reagent Co., Ltd. Methyl laurate (>98%) was purchased from Shanghai Titan Scientific Co., Ltd. Tetrahydronaphthalene and alkane standards (n-C6 to C26) were purchased from Aladdin. Refined palm oil, duck fat, soybean oil was obtained from a local market in Qingdao City, China. All the above chemicals were used without further purification. Waste cooking oil was provided by a local waste oil recycling station in Qingdao City, China, and washed several times with water before use to remove the water-soluble substances, e.g., metal ions and salts. Maize straw (*ms*) was collected from a farmland in Zibo City, China. After removing the leaves and skin, the *ms* was cut into sections with length of ~8.0 mm, washed with HCl (1 M) for 2 times to remove the inherent metal species and then with water until neutral pH, finally freeze-dried for further use. Deionized water was used in all experiments.

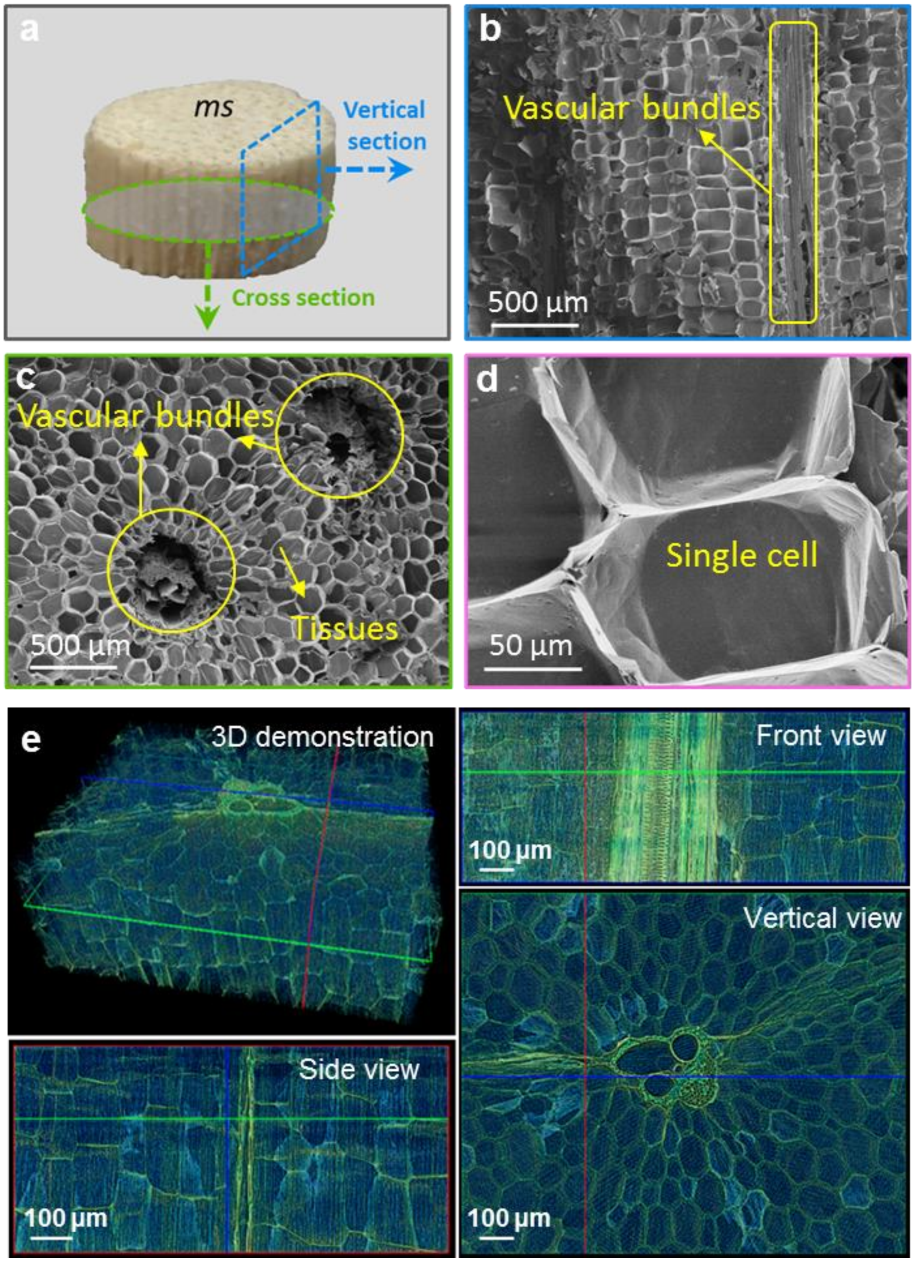


**Supplementary Fig. 1.** (**a**) Photograph, (**b-d**) SEM and (**e**) Synchrotron X-ray tomographic images of *ms*-template.

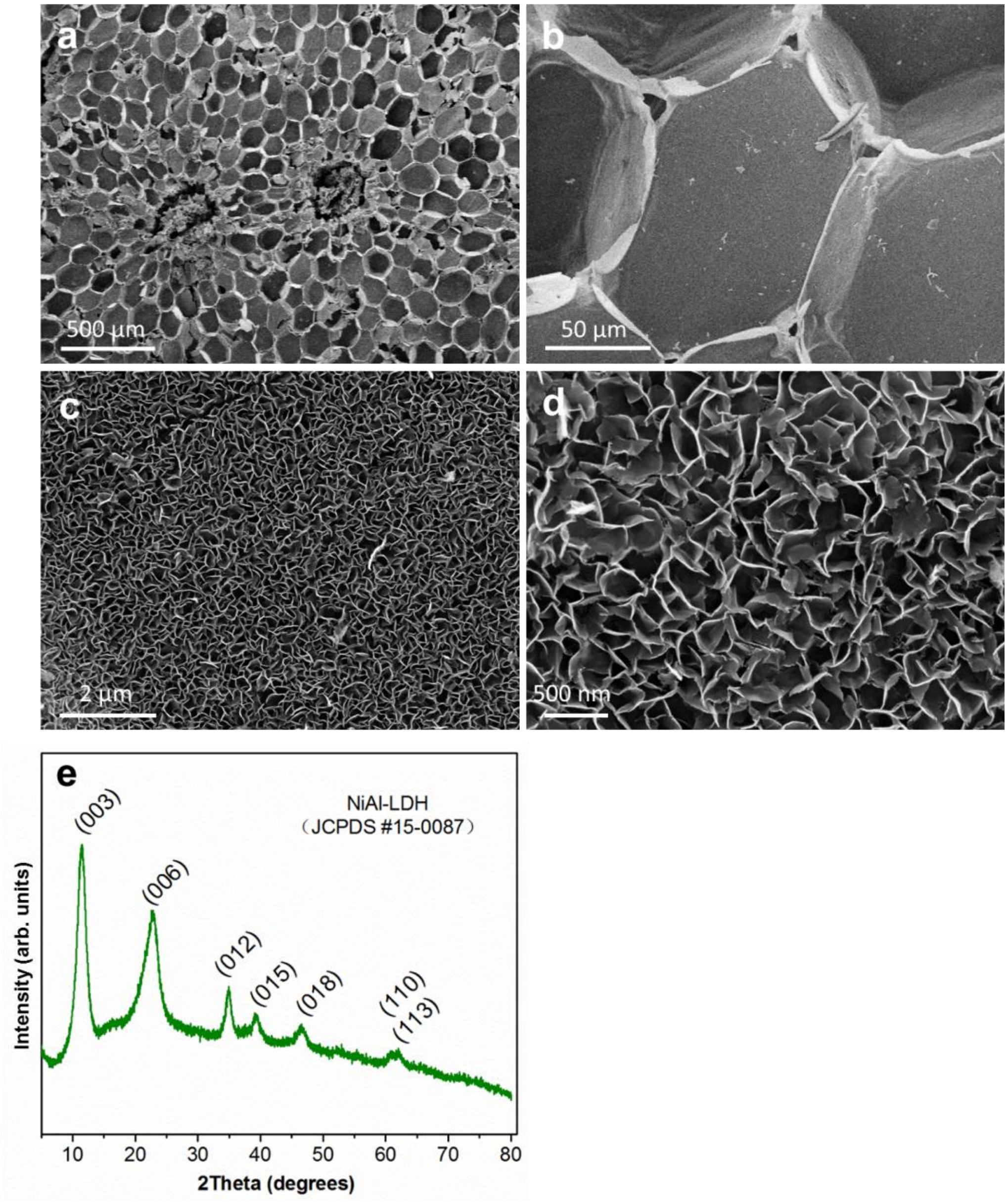


**Supplementary Fig. 2.** (**a-d**) SEM images of *ms*-NiAl-LDHs, showing the biological structure and the NiAl-LDHs nanosheets on the cell wall. (**e**) XRD result of *ms*-NiAl-LDHs, showing the peaks that belong to NiAl-LDHs.

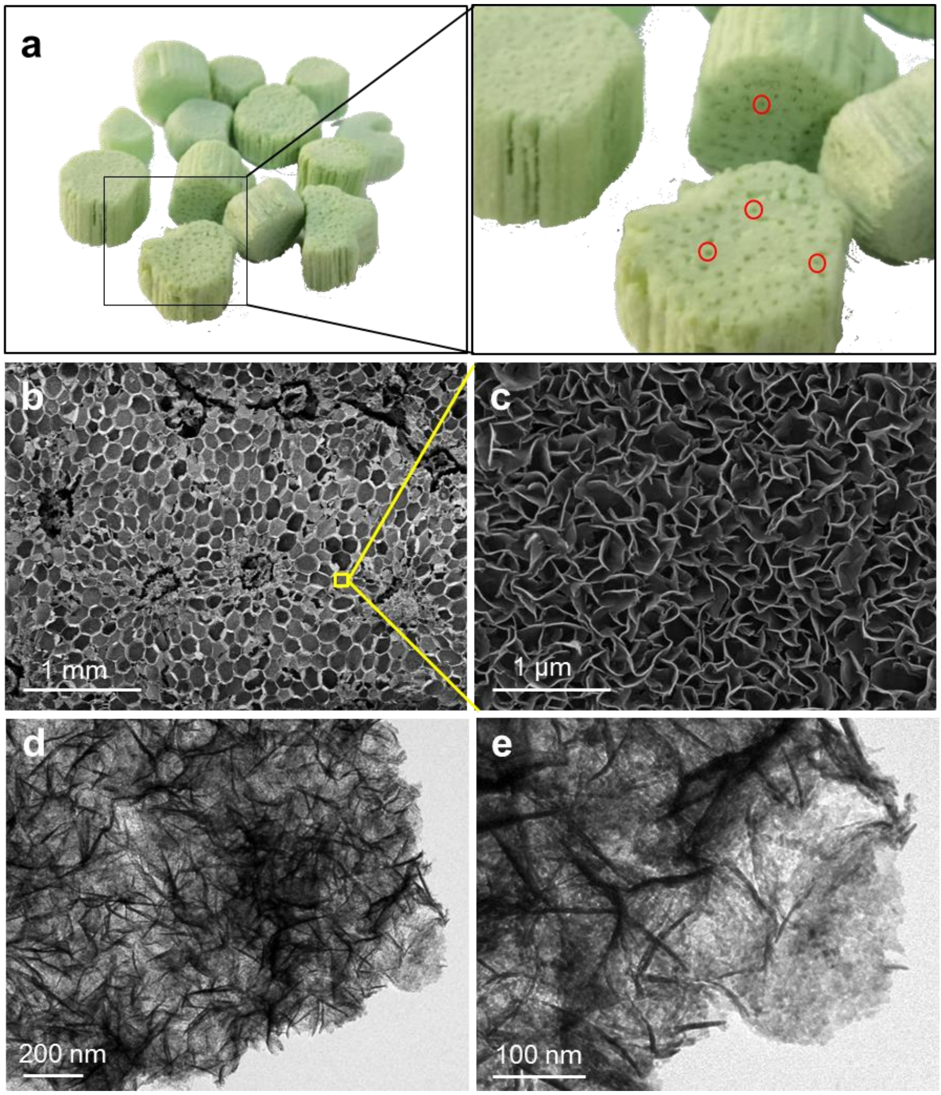


**Supplementary Fig. 3.** (**a**) Photographs, (**b, c**) SEM and (**d, e**) TEM images of *ms*-NiAl, showing the biological architecture completely replicated from *ms*-template and the nanosheet structure derived from LDHs.

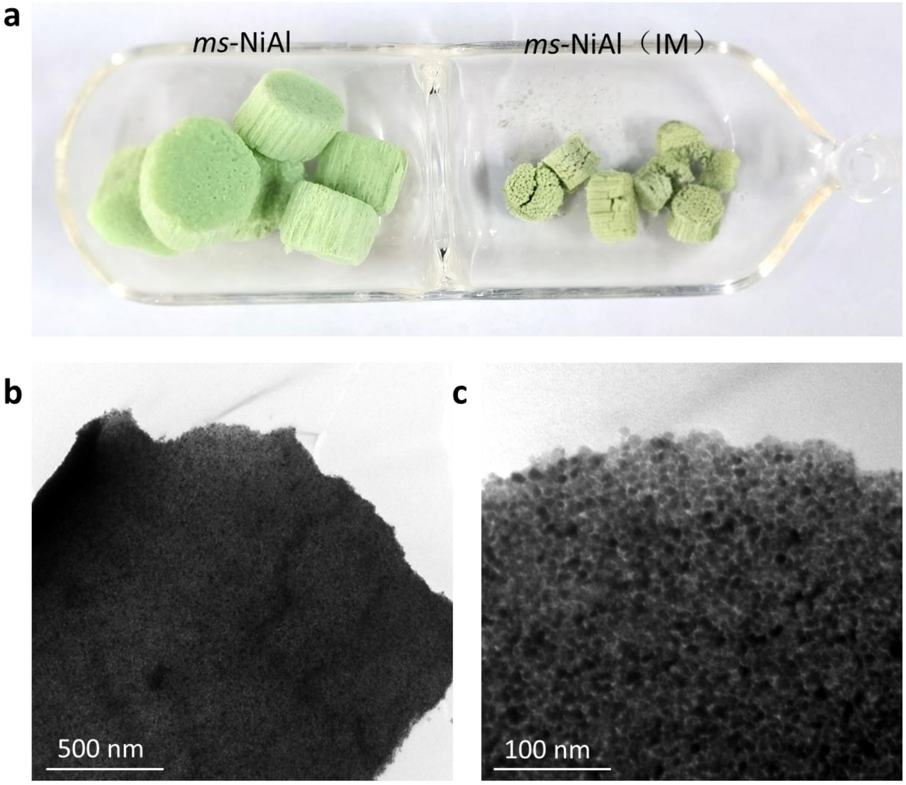


**Supplementary Fig. 4.** (**a**) Photograph and (**b**, **c**) TEM images of *ms*-NiAl (IM) prepared by the impregnation method.

*Synthesis of ms-NiAl(IM):* The *ms* was soaked in the solution containing 4.5 mmol of $Ni(NO_3)_2$ $6H_2O$, 1.5 mmol of $Al(NO_3)_3$ $9H_2O$, 13.5 mmol of urea and 90 mL of water. After saturated adsorption, the monolithic sample was freeze-dried and calcined under air at 800 ℃ for 1 h to obtain the *ms*-NiAl (IM).

However, replicating the *ms* structure by the simple impregnation method was not successful as incomplete replication with severe shrinkage was observed, which highlights the importance of hydrothermal process towards fabrication of metal hydroxides nanosheets for replication of the *ms* structure.

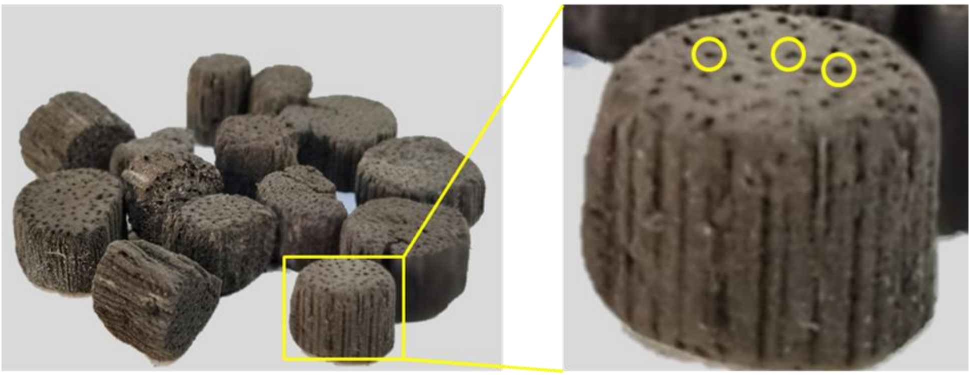

**Supplementary Fig. 5.** Photographs of *ms*-NiAl-P350, showing the biological architecture replicated from *ms*-template.

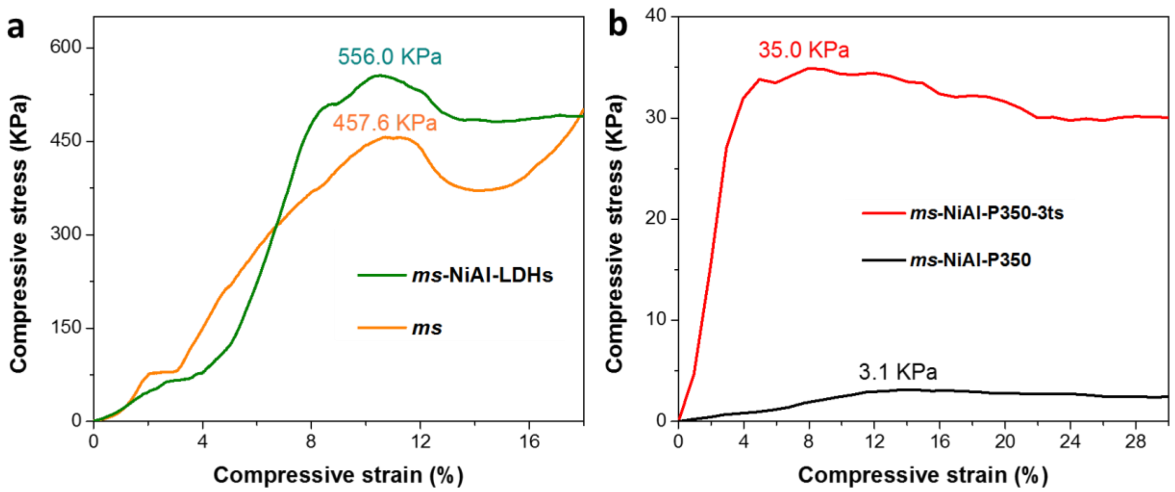


**Supplementary Fig. 6.** Compressive stress-strain curves: (**a**) *ms* and *ms*-NiAl-LDHs, (**b**) *ms*-NiAl-P350 and *ms*-NiAl-P350-3ts (repeating NiAl-LDHs growth (i.e., hydrothermal process) for 3 times). The compressive strength of *ms*-NiAl-P350 decreased after removing the *ms*-template, but can be enhanced by repeating NiAl-LDHs growth during synthesis.

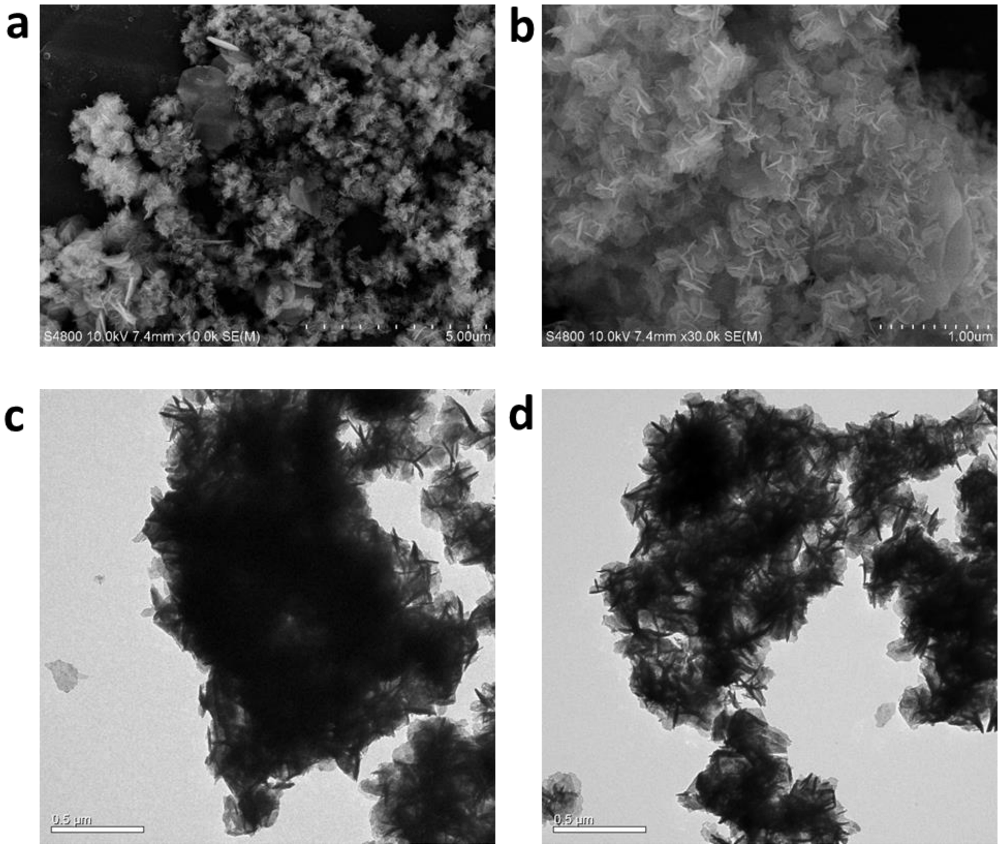


**Supplementary Fig. 7.** (**a, b**) SEM and (**c, d**) TEM images of NiAl-P350 powder synthesized without *ms*-template.

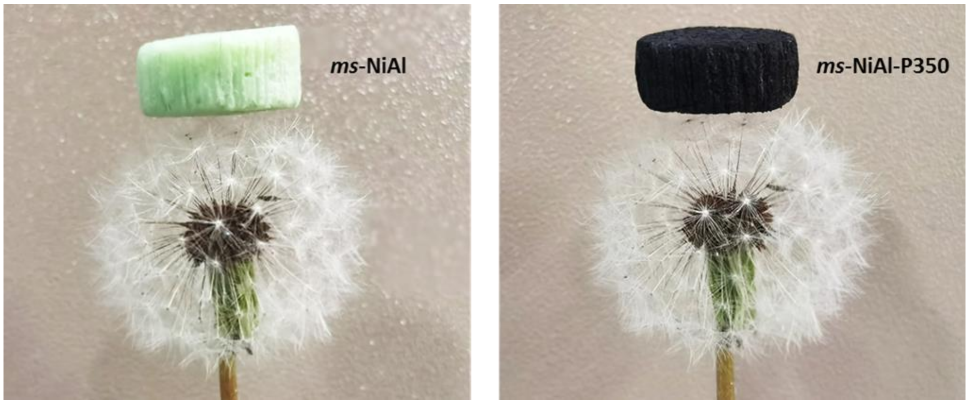


**Supplementary Fig. 8.** Photographs of *ms*-NiAl (left) and *ms*-NiAl-P350 (right) on dandelions, implying their low density.

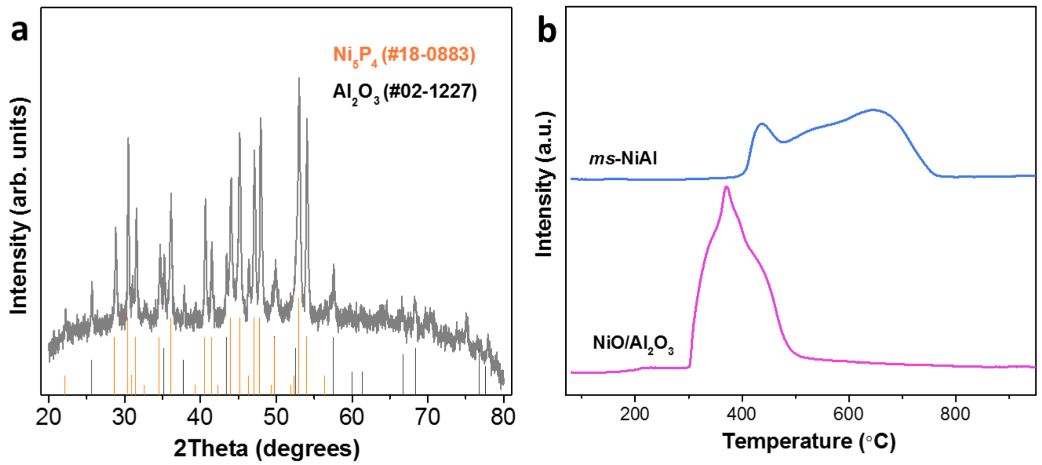


**Supplementary Fig. 9. (a)** XRD result of the $NiO/Al_2O_3$ after phosphorizing at 350 °C using $NaH_2PO_2$ as P source ($NiO/Al_2O_3$-P350), showing the formation of $Ni_5P_4$ phase. **(b)** $H_2$-TPR curves of *ms*-NiAl and $NiO/Al_2O_3$. The $H_2$ consumption peak of *ms*-NiAl shifts towards higher temperature compared with that of $NiO/Al_2O_3$, indicating that the interaction between NiO and $Al^{3+}$ species in *ms*-NiAl can stabilize the NiAl mixed oxide phase against $H_2$ reduction.

*Synthesis of $NiO/Al_2O_3$ and $NiO/Al_2O_3$-P350*: Firstly, 1.5 mmol of $Al(NO_3)_3$ $9H_2O$ and 13.5 mmol of urea were dissolved in 90 mL of water. Then, the solution was transferred into a teflon-lined stainless-steel autoclave, sealed, heated up to 100 °C and kept at that temperature for 24 h. The product was collected by filtration, washed with water, dried and calcined under air at 800 ℃ for 1 h to obtain the $Al_2O_3$. Next, the as-obtained $Al_2O_3$ was dispersed in a solution containing 4.5 mmol of $Ni(NO_3)_2$ $6H_2O$. After evaporation, the solid product was calcined at 800 ℃ for 1 h to obtain the $NiO/Al_2O_3$, and subsequently phosphorized according to the procedure similar to that of *ms*-NiAl-P350 to obtain the $NiO/Al_2O_3$-P350.

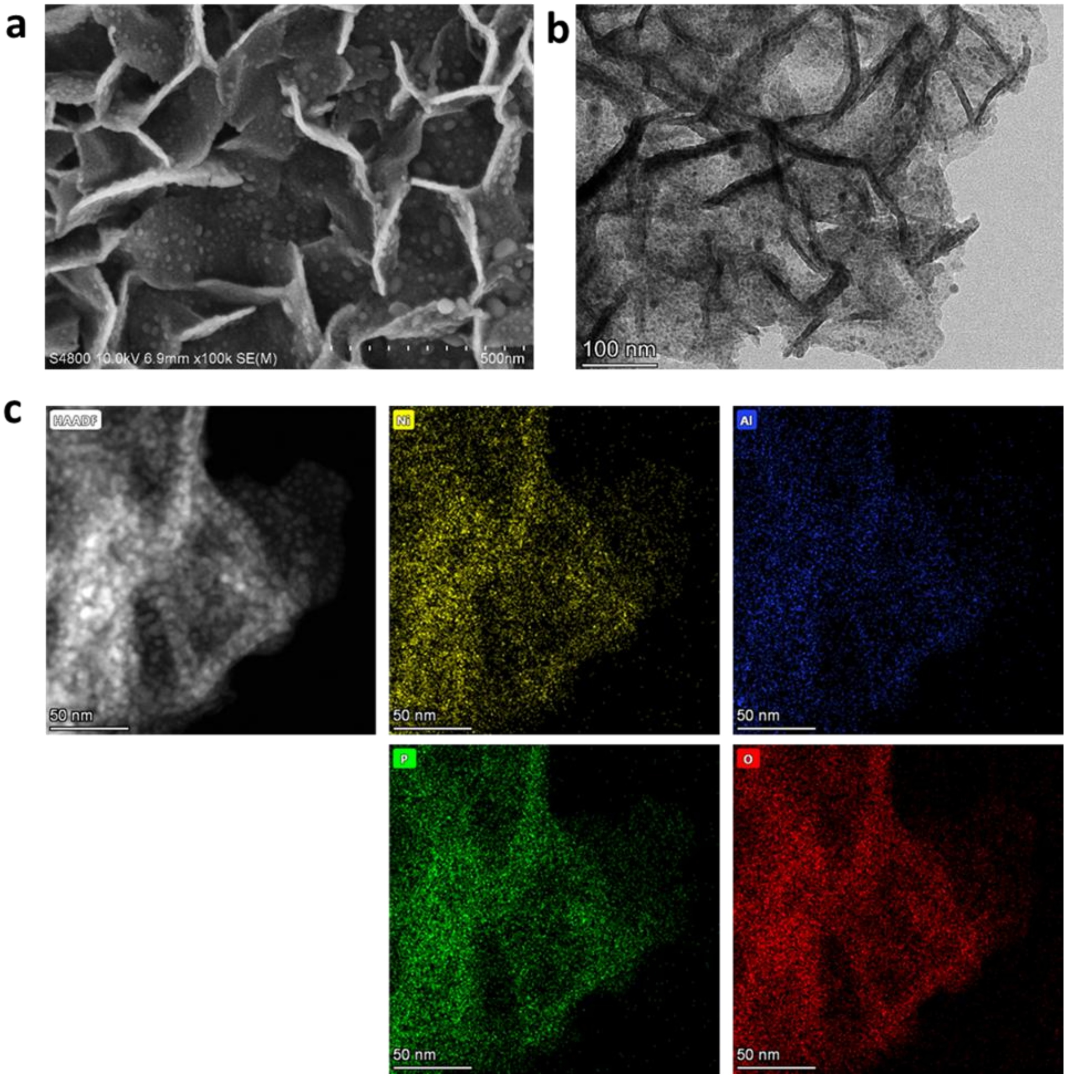


**Supplementary Fig. 10.** (**a**) SEM, (**b**) TEM and (**c**) HAADF-STEM and the corresponding EDS elemental mapping images of *ms*-NiAl-P450.

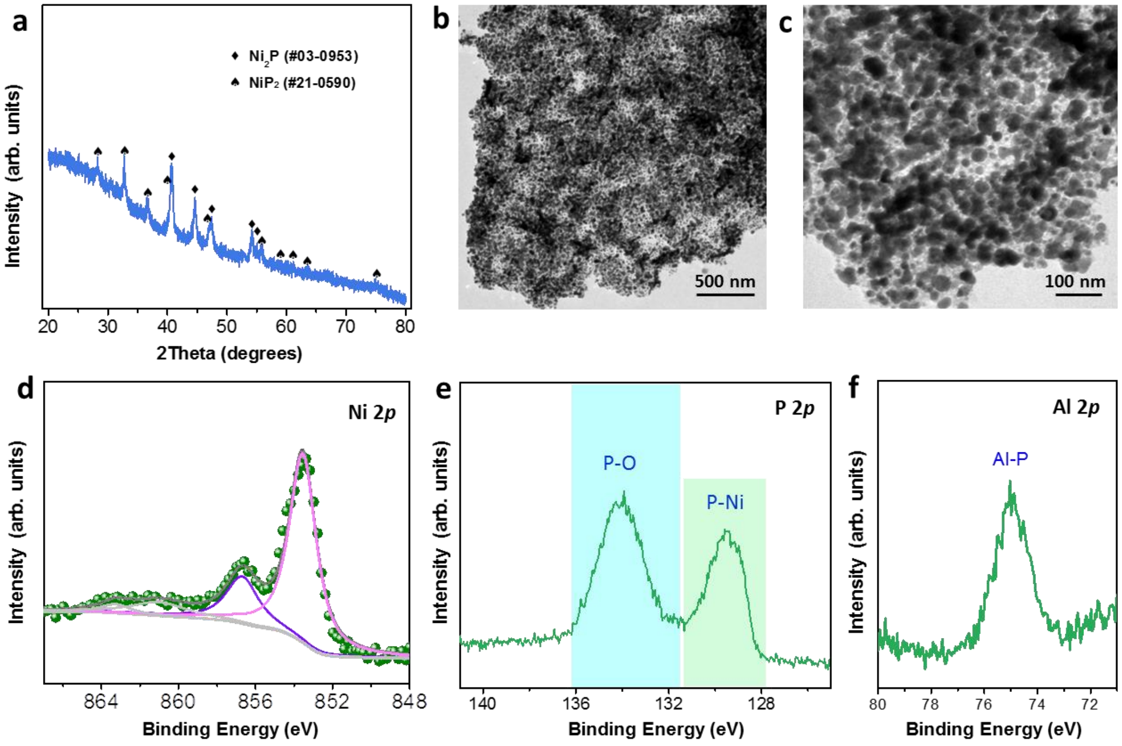


**Supplementary Fig. 11.** (**a**) XRD curve, (**b, c**) TEM images and (**d-f**) XPS results of *ms*-NiAl-$H_2$800-P350.

*Synthesis of ms-NiAl-$H_2$800-P350*: The *ms*-NiAl was reduced at 800 ℃ for 1 h with a heating rate of 10 ℃/min under $H_2$/Ar (10%/90%) flow, to break the interaction between NiO and $Al^{3+}$. After cooling down to room temperature, the sample was passivated in a flow of 0.5% $O_2$ in Ar (100 mL/min) for 2 h. Then, the as-obtained sample was phosphorized at 350 ℃ using the same procedure with that of *ms*-NiAl-P350. The final sample was marked as *ms*-NiAl-$H_2$800-P350.

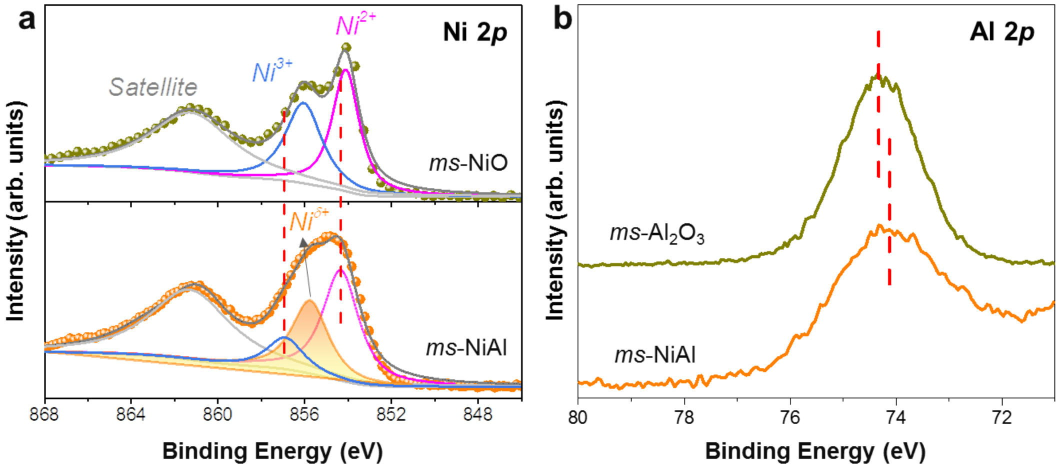


**Supplementary Fig. 12. (a)** XPS Ni 2*p* spectra of *ms*-NiAl and *ms*-NiO. **(b)** XPS Al 2*p* spectra of *ms*-NiAl and *ms*-$Al_2O_3$.

*Synthesis of ms-NiO* and *ms-$Al_2O_3$:* The *ms*-NiO was prepared with the same procedure as the *ms*-NiAl, except for the absence of Al precursor. The *ms*-$Al_2O_3$ was prepared with the same procedure as the *ms*-NiAl, expect for the absence of Ni precursor.

The Ni $2p_{3/2}$ XPS spectrum of *ms*-NiO (Supplementary Fig. 12a and Table 4) showed two peaks at 855.9 eV and 854.0 eV, which were assigned to $Ni^{3+}$and $Ni^{2+}$, respectively. In the case of *ms*-NiAl, a new peak belonging to $Ni^{\delta+}$ peak ($2<\delta<3$) appeared at 855.7 eV, while the peaks of $Ni^{3+}$ (856.9 eV) and $Ni^{2+}$ (854.3 eV) both shifted to higher binding energy. In Al 2*p* region (Supplementary Fig. 12b), the binding energy of *ms*-NiAl (74.1 eV) shifted to a lower value compared to that of *ms*-$Al_2O_3$ (74.3 eV). These results illustrate that the interaction between $Al^{3+}$ and NiO was constructed in *ms*-NiAl, wherein electrons transfer from Ni-O to the nearby O-Al.

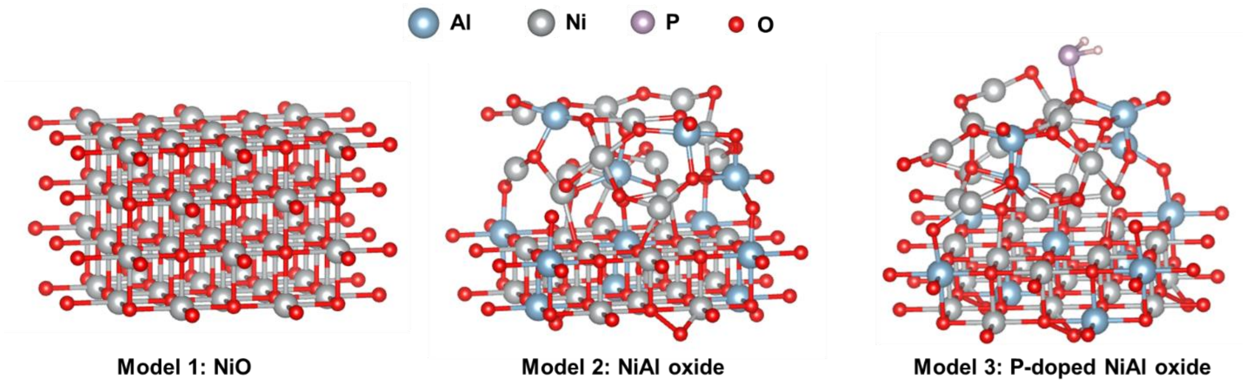


**Supplementary Fig. 13.** Catalyst models used for DFT calculations in this study.

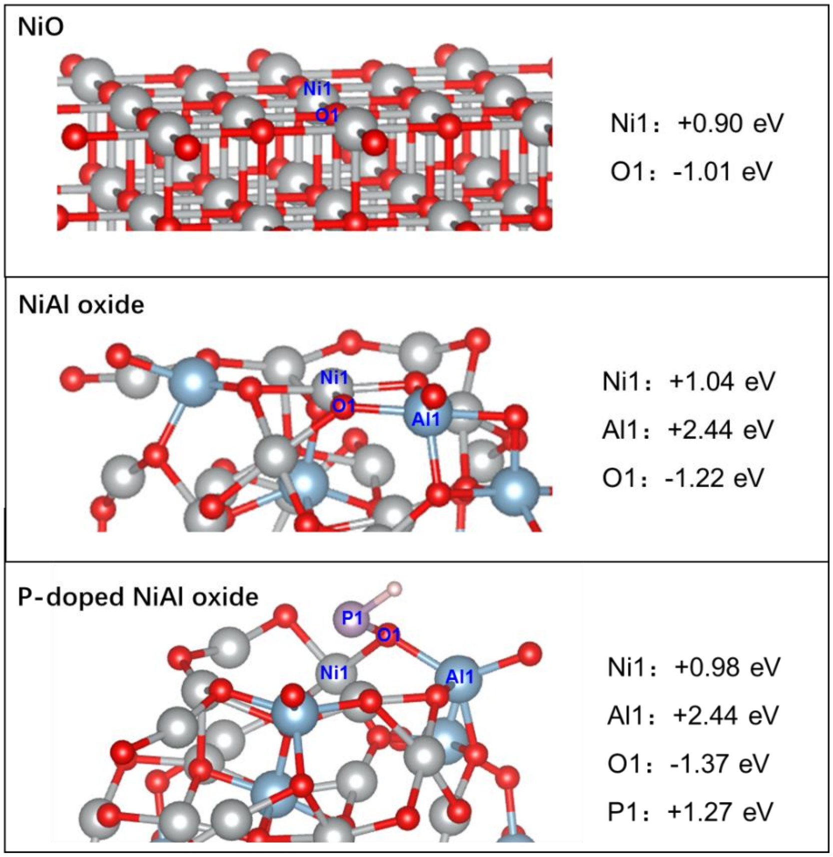


**Supplementary Fig. 14.** The Bader charges of typical Ni, O, Al or P atoms in NiO, NiAl oxide and P-doped NiAl-oxide models.

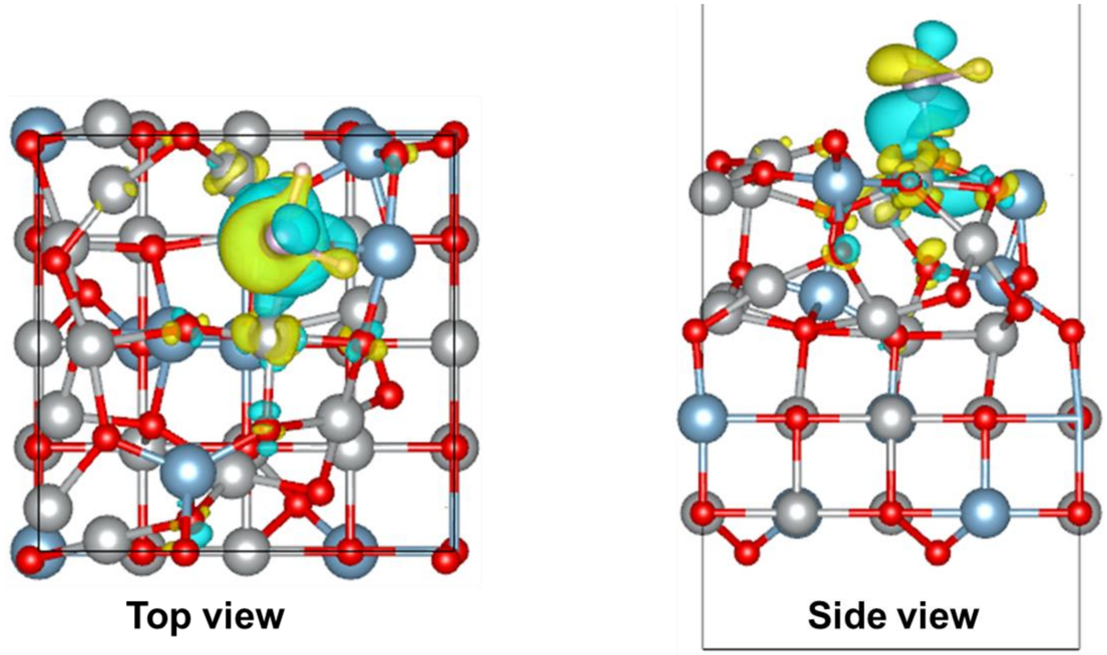


**Supplementary Fig. 15.** The differential charge density distribution of P doping in the NiAl oxide. (Yellow isosurface means electron accumulation, blue isosurface means electron depletion)

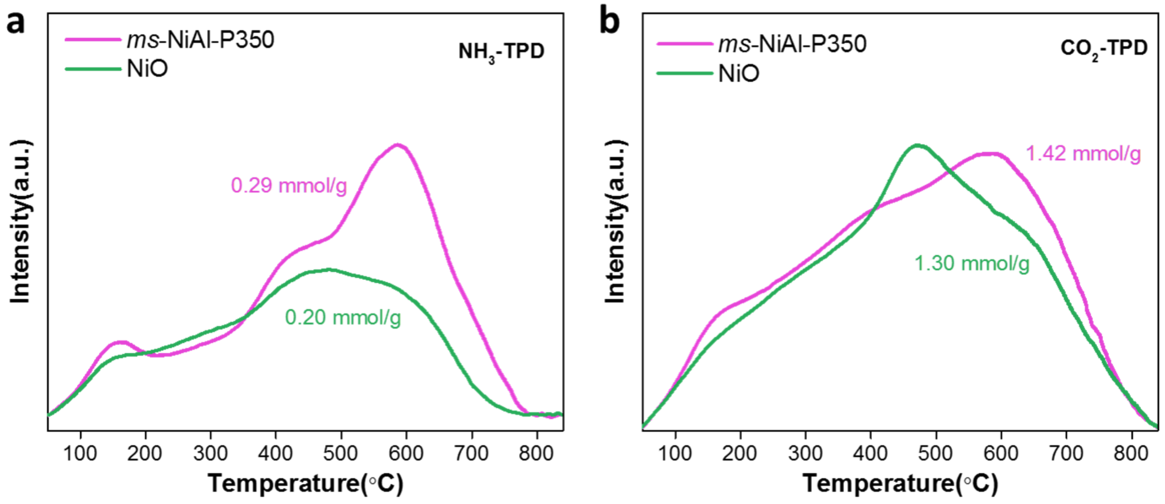


**Supplementary Fig. 16.** The $NH_3$-TPD (**a**) and $CO_2$-TPD (**b**) profiles of the *ms*-NiAl-P350 and NiO, verifying the presence of acid and base sites.

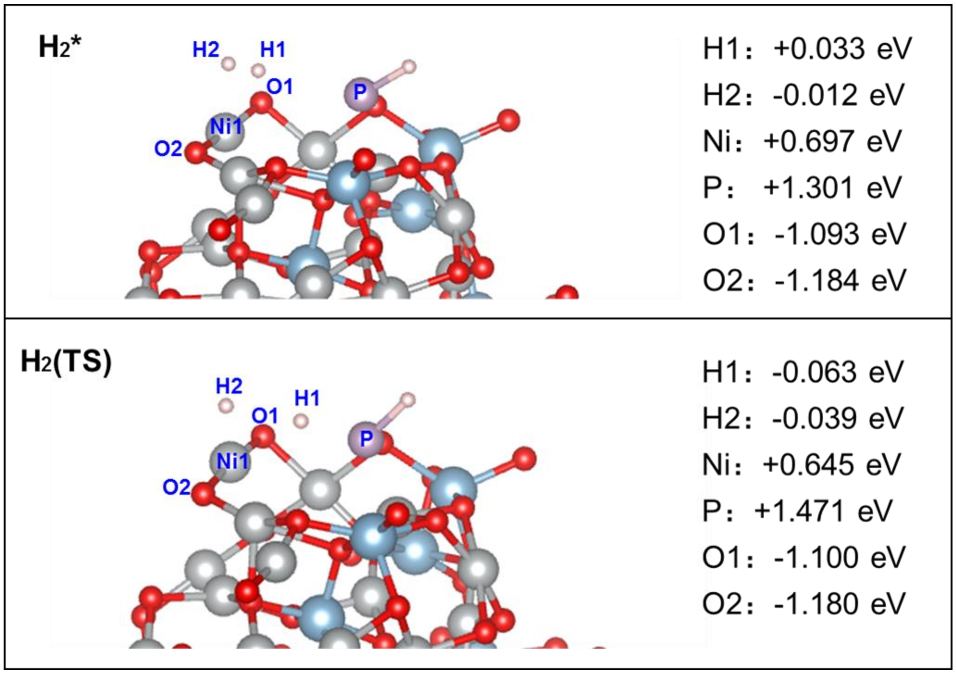


**Supplementary Fig. 17.** The Bader charges of typical Ni, O, Al or P atoms before and after $H_2$ activation on the P-doped NiAl-oxide model.

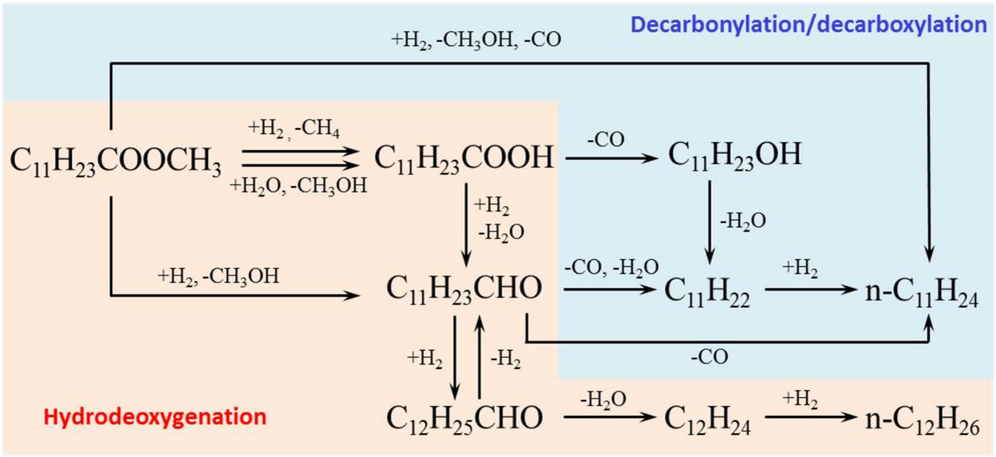


**Supplementary Fig. 18.** Proposed pathways for hydrotreating of methyl laurate.

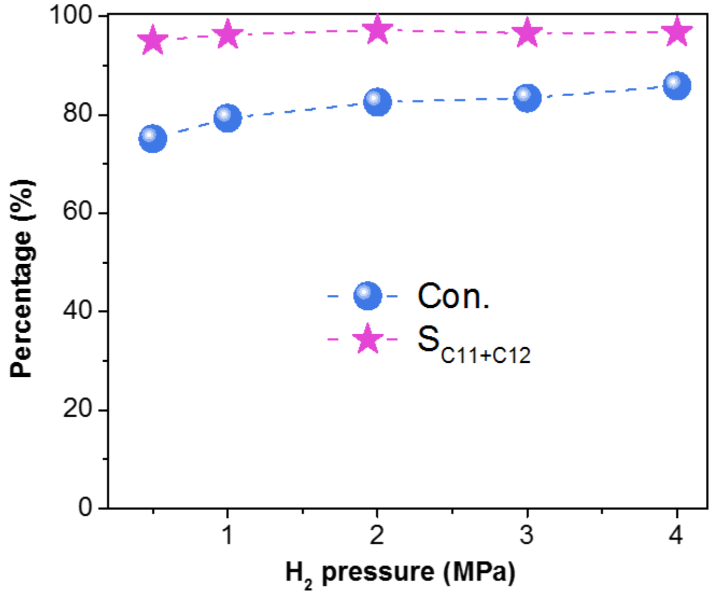


**Supplementary Fig. 19.** Conversion of methyl laurate and selectivity towards $C_{11}H_{24}$/$C_{12}H_{26}$ as function of $H_2$ pressure over *ms*-NiAl-P350. Reaction conditions: T=350 °C, WHSV=28.3 $h^{-1}$.

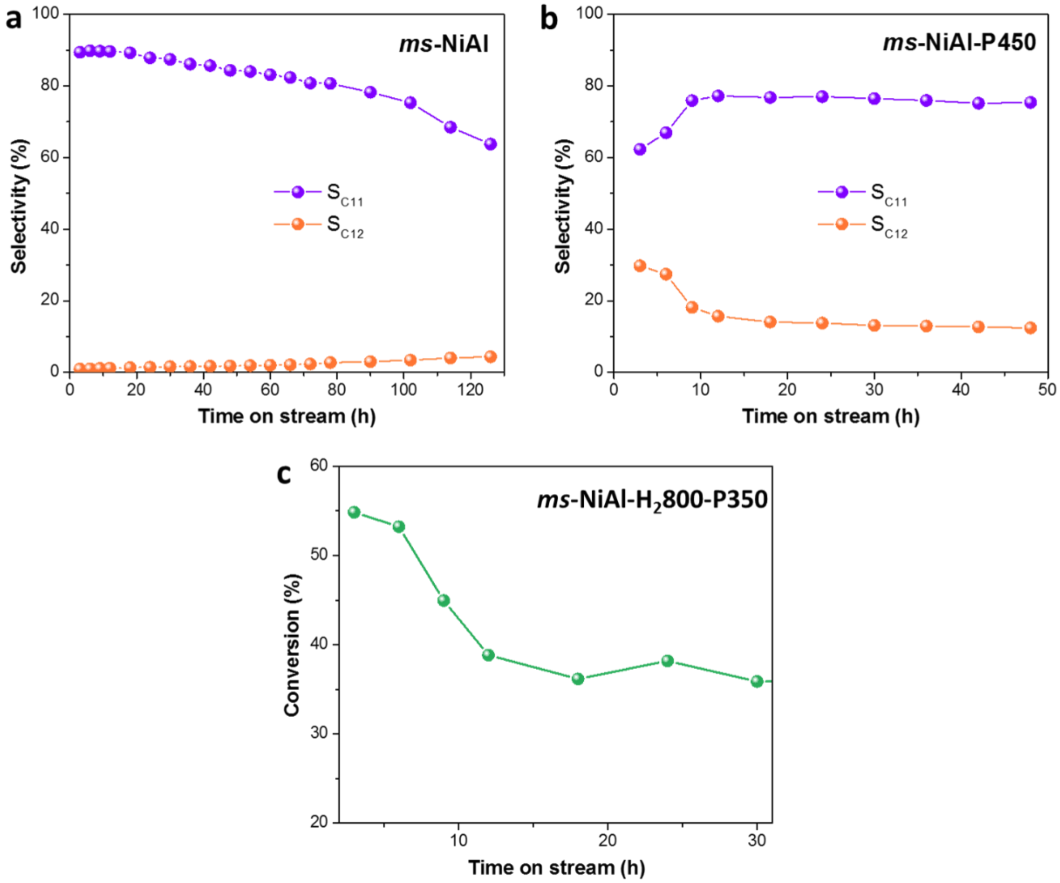


**Supplementary Fig. 20.** Selectivity towards $C_{11}H_{24}$ and $C_{12}H_{26}$ over **(a)** *ms*-NiAl and **(b)** *ms*-NiAl-P450, respectively. **(c)** Conversion of methyl laurate over *ms*-NiAl-$H_2$800-P350. Reaction conditions: T=350 ℃, $P_{H2}$=3.0 MPa, WHSV=28.3 $h^{-1}$.

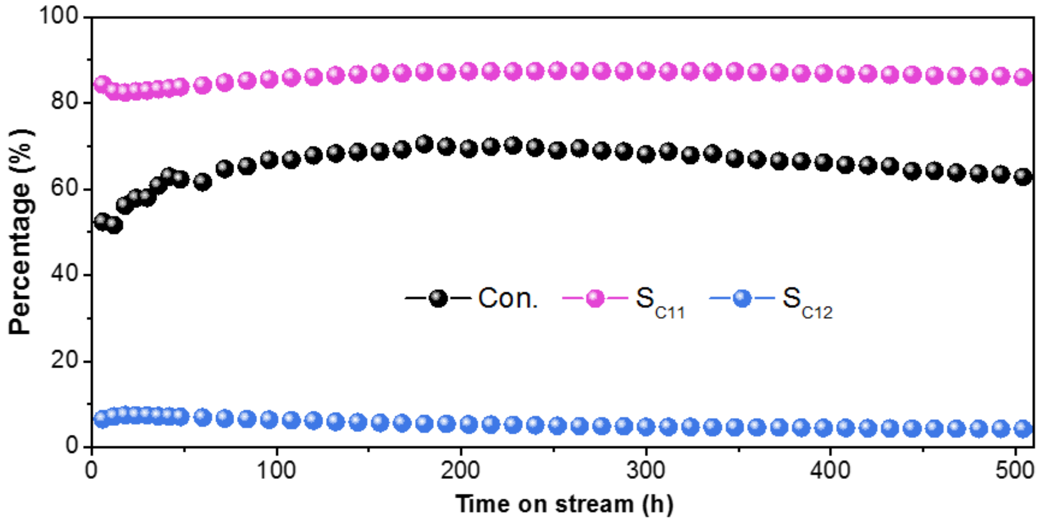


**Supplementary Fig. 21.** Conversion of methyl laurate and selectivity towards $C_{11}H_{24}$/$C_{12}H_{26}$ as function of time over NiAl-P350. Reaction conditions: T=350 ℃, $P_{H2}$=3.0 MPa, WHSV=28.3 $h^{-1}$.

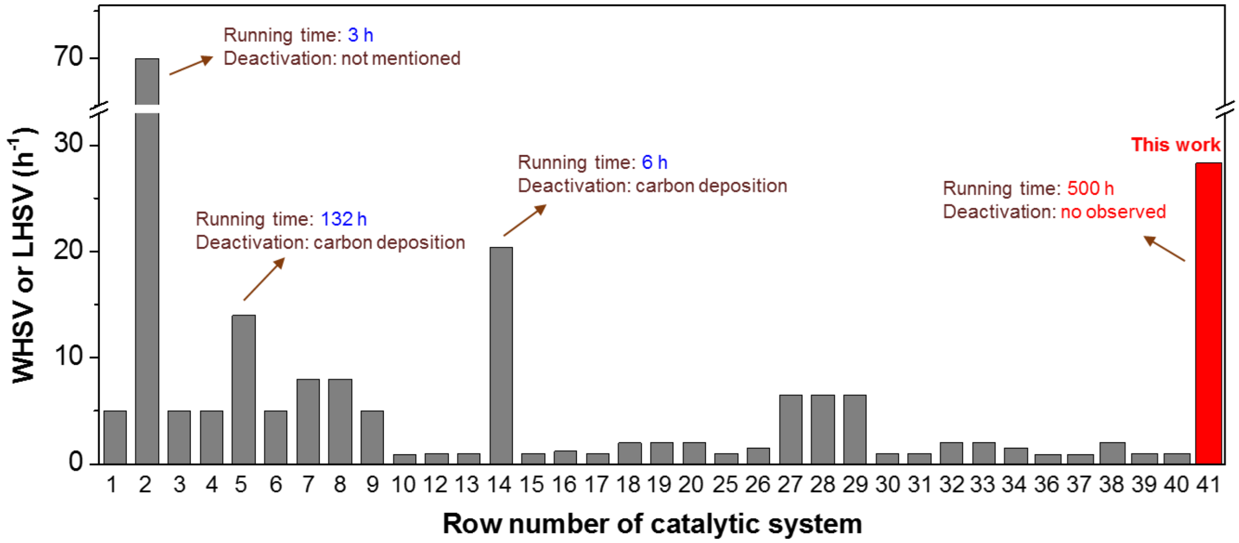


**Supplementary Fig. 22.** WHSV or LHSV of different catalytic systems. Numbers in X-axis correspond to the row numbers in Supplementary Table 6.

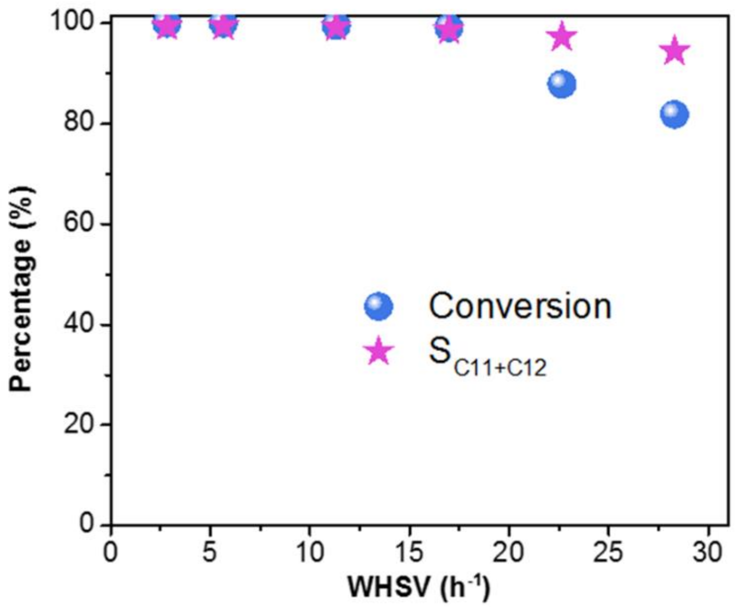


**Supplementary Fig. 23.** Conversion of methyl laurate and selectivity towards $C_{11}H_{24}/C_{12}H_{26}$ as function of WHSV value over *ms*-NiAl-P350. Reaction conditions: T=350 ℃, $P_{H2}$=3.0 MPa.

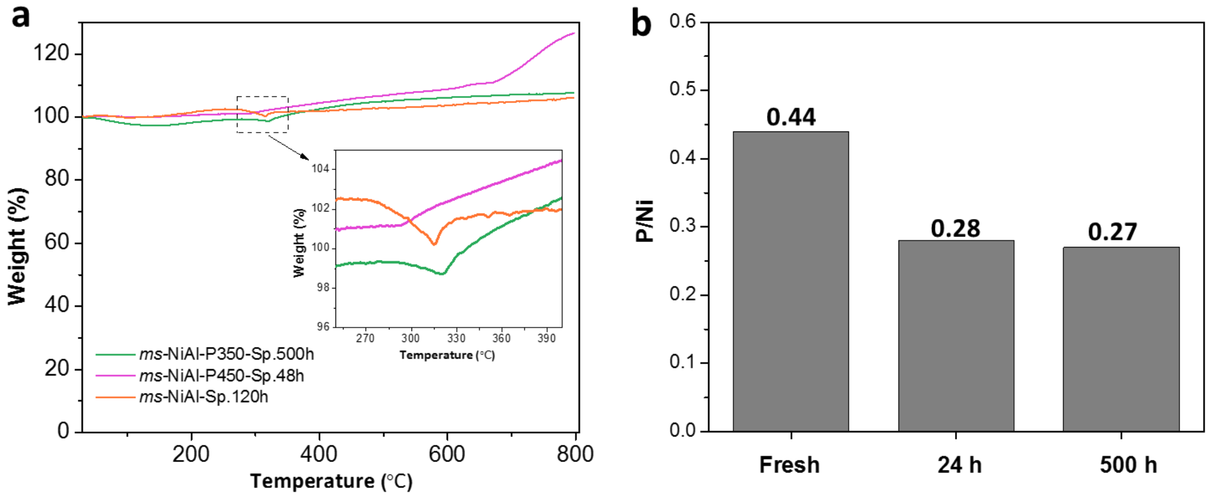


**Supplementary Fig. 24.** **(a)** The TGA curves of the spent catalysts, showing no obvious weight loss assigned to coke deposition for *ms*-NiAl-P350-Sp.500h. **(b)** Atomic ratios of P/Ni for fresh and spent *ms*-NiAl-P350 determined by ICP-OES. After initial P loss (due to the desorption of surface P species) in 24 h, the P content is almost unchanged during 500 h of operation.

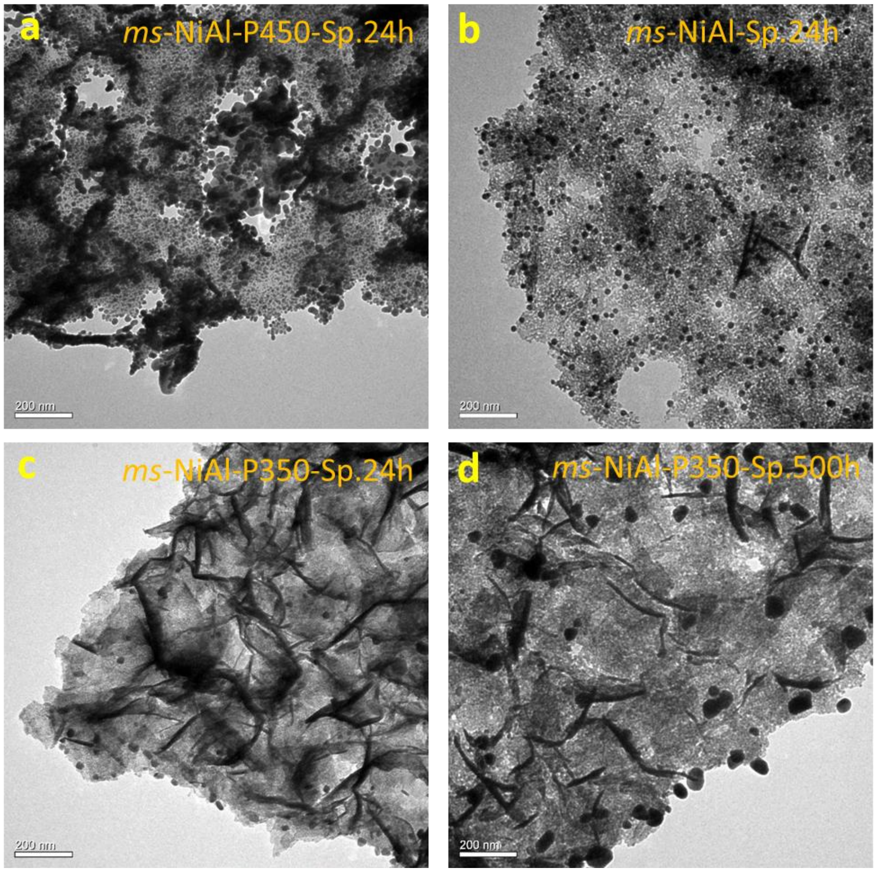


**Supplementary Fig. 25.** TEM images of the spent catalysts : (**a**) *ms*-NiAl-P450-Sp.24h, (**b**) *ms*-NiAl-Sp.24h, (**c**) *ms*-NiAl-P350-Sp.24h, (**d**) *ms*-NiAl-P350-Sp.500h.

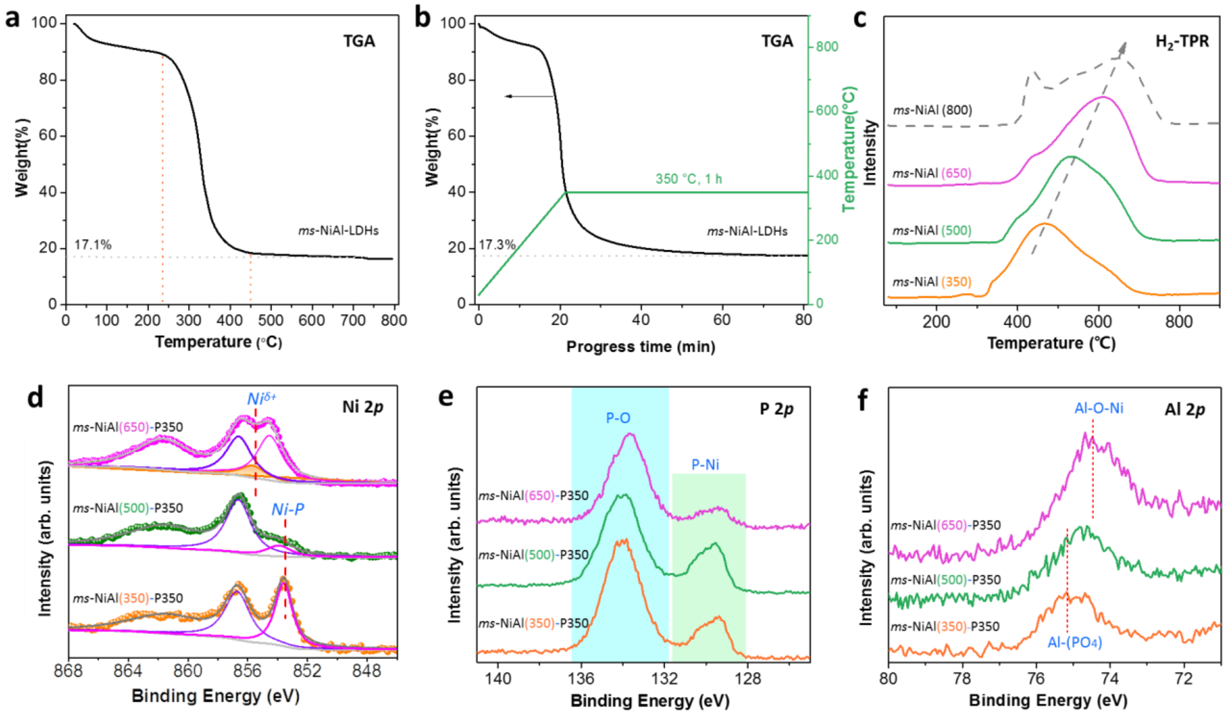


**Supplementary Fig. 26.** (**a,b**) TGA curves of *ms*-NiAl-LDHs: (a) weight loss as function of temperature under air flow at the heating rate of 10 ℃/min; (b) weight loss as function of time under air flow with temperature holding at 350 ℃. The results show that the organic matters in *ms*-NiAl-LDHs can be completely removed after calcination at 350 ℃ for 1 h. (**c**) $H_2$-TPR curves of *ms*-NiAl(t) (t represents the calcination temperature). The $H_2$ consumption peaks shift towards higher temperature along with increasing the calcination temperature, indicating the enhanced interaction between $Al^{3+}$ species and NiO. (**d-f**) The XPS results of *ms*-NiAl(t)-P350.

*Discussion about the effect of calcination temperature:* Based on the results of XRD, XPS, XAFS, DFT calculation and catalytic tests, we inferred that fabricating the *ms*-NiAl ($Al^{3+}$-doped NiO-type NiAl-mixed oxide) by calcination at 800 ℃ under air was critical for the formation of P-doped NiAl-oxide. To verify this inference, we attempted to adjust the interaction of Ni-O-Al by changing the calcination temperature. According to the TGA curves of *ms*-NiAl-LDHs (Supplementary Fig. 26a-b), the organic matters present in *ms*-NiAl-LDHs can be completely removed after calcination at 350 ℃ for 1 h. Therefore, three samples of *ms*-NiAl(t) (t represents the calcination temperature) were prepared by calcination at 350 ℃, 500 ℃, and 650 ℃, respectively. The $H_2$-TPR profiles (Supplementary Fig. 26c) show that increasing the calcination temperature leads to a higher initial reduction temperature for *ms*-NiAl(t). Moreover, the XPS results show that increasing the calcination temperature leads to an enhancement of $Ni^{\delta+}$ peak (Supplementary Fig. 26d), a weakening of Ni-P peak (Supplementary Fig. 26e) and a transformation of Al species from $Al^{3+}$ to Al-O-Ni (Supplementary Fig. 26f). These observations demonstrate that the Ni-O-Al interaction is enhanced by elevating the calcination temperature, which is beneficial for the formation of a P-doped NiAl-oxide structure instead of nickel phosphide.

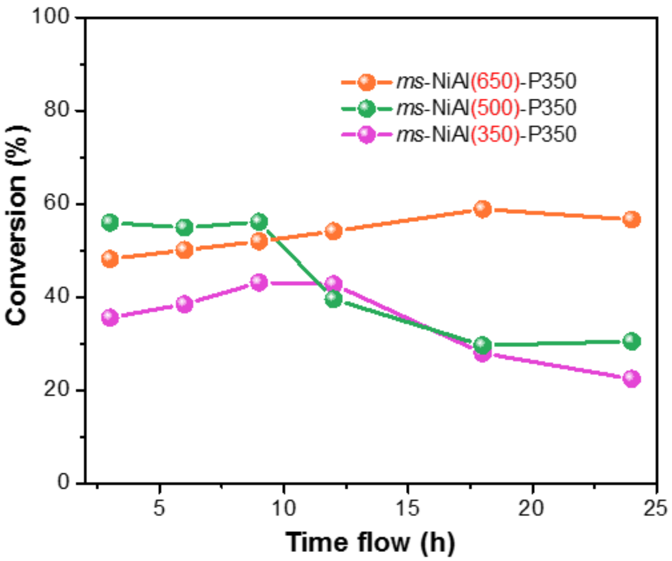


**Supplementary Fig. 27.** Methyl laurate conversion as function of time over *ms*-NiAl(t)-P350. Reaction conditions: T=350 ℃, P=3.0 MPa, WHSV=28.3 $h^{-1}$. As expected, the *ms*-NiAl(350)-P350 and *ms*-NiAl(500)-P350 were deactivated within a period of 24 h, while *ms*-NiAl(650)-P350 exhibited a stable activity during this period, but with a lower conversion compared to that of *ms*-NiAl(800)-P350.

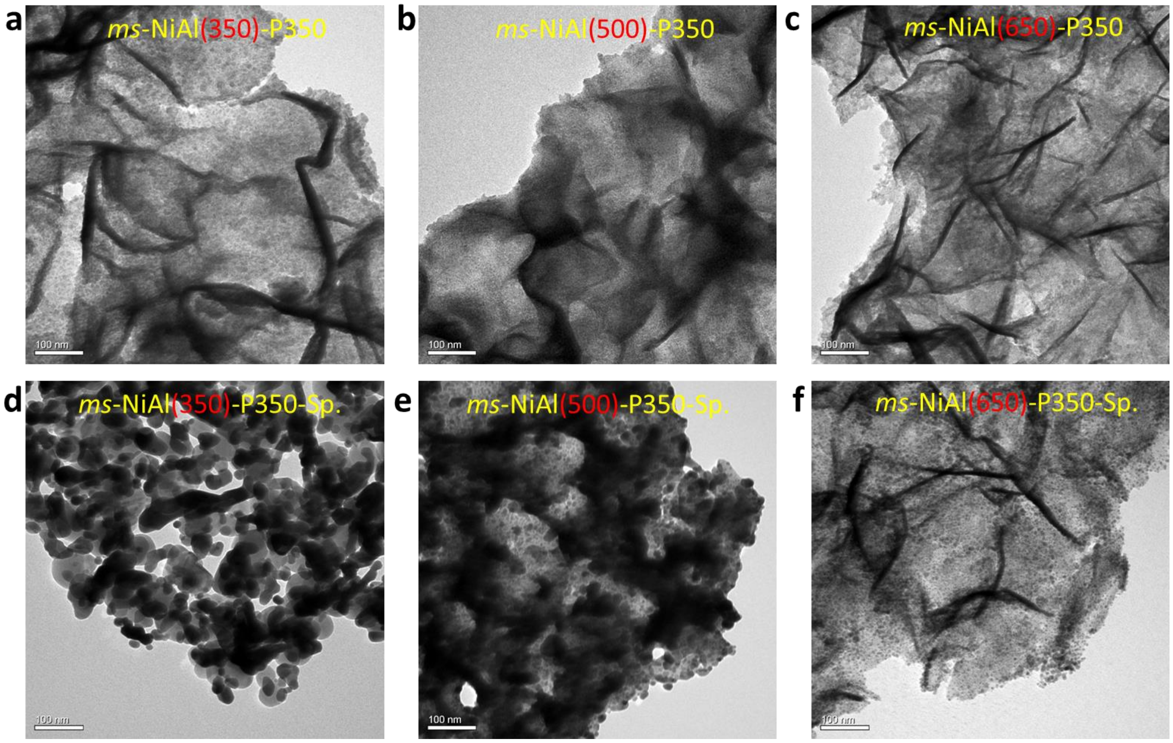


**Supplementary Fig. 28.** (**a-c**) TEM images of *ms*-NiAl(t)-P350. (**d-f**) TEM images of the *ms*-NiAl(t)-P350 after using for 24 h. These results indicate that a lower calcination temperature leads to serious metal agglomeration in catalysis, due to the weaker interaction between NiO and $Al^{3+}$.

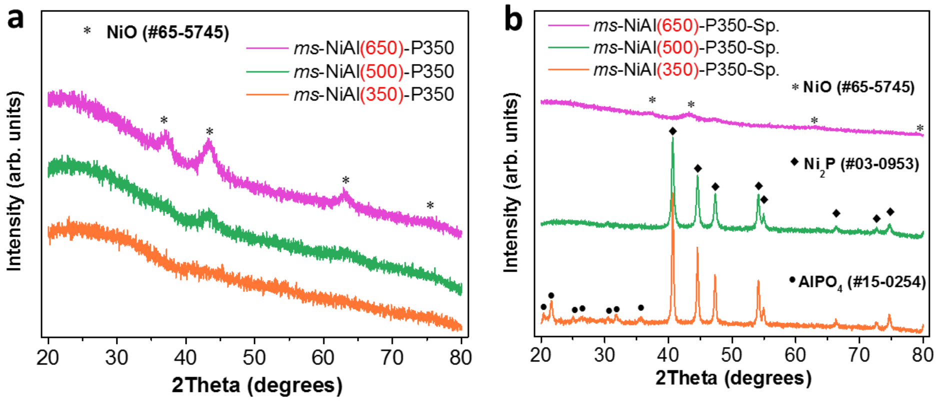


**Supplementary Fig. 29.** XRD results of the **(a)** fresh and **(b)** spent *ms*-NiAl(t)-P350. $Ni_2P$ nanoparticles were formed in both the *ms*-NiAl(350)-P350-Sp.24h and *ms*-NiAl(500)-P350-Sp.24h. However, there was no obvious change in crystalline structure for the *ms*-NiAl(650)-P350-Sp.24h. These results further confirm that the enhancement of catalyst stability in hydrotreating process can be achieved by increasing the interaction between NiO and $Al^{3+}$.

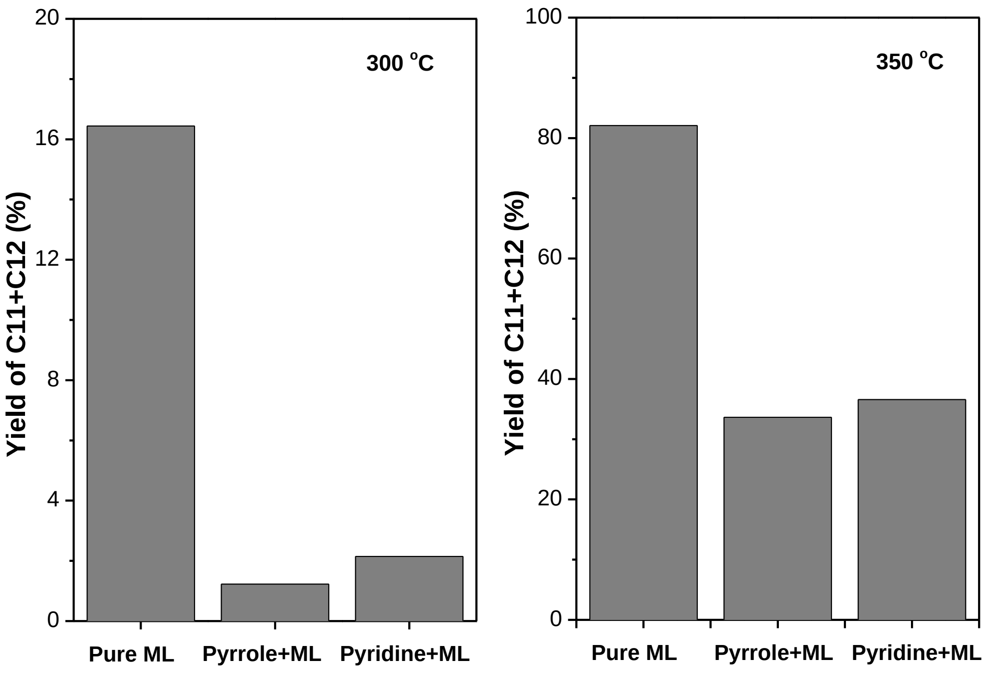


**Supplementary Fig. 30.** Influences of molecular Lewis-base (pyridine) and Lewis-acid (pyrrole) on the catalytic activity of Ni(-O-Al) ··P FLP for hydrotreating methyl laurate (ML) to $C_{11}H_{24}$ and $C_{12}H_{26}$. Reaction conditions: $P_{H2}$=3.0 MPa, WHSV=28.3 $h^{-1}$. The content of pyrrole/pyridine in the feedstock is 20 wt.%.

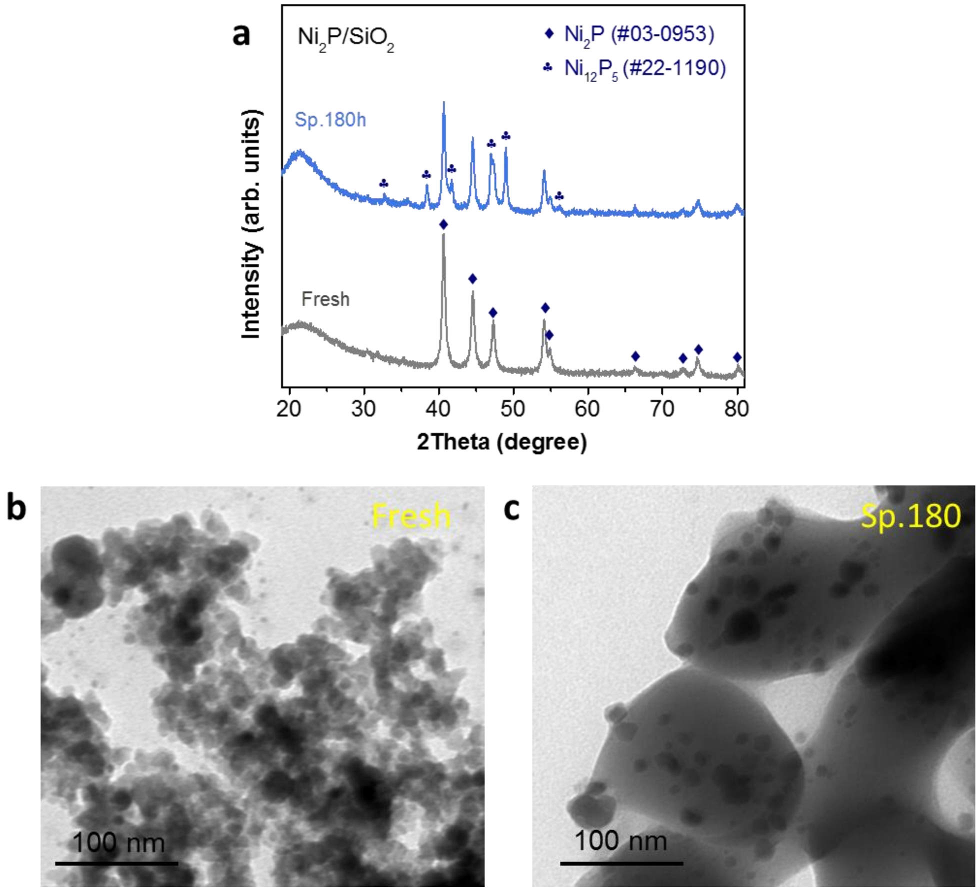


**Supplementary Fig. 31.** (**a**) XRD patterns and (**b** and **c**) TEM images of the fresh and spent $Ni_2P/SiO_2$.

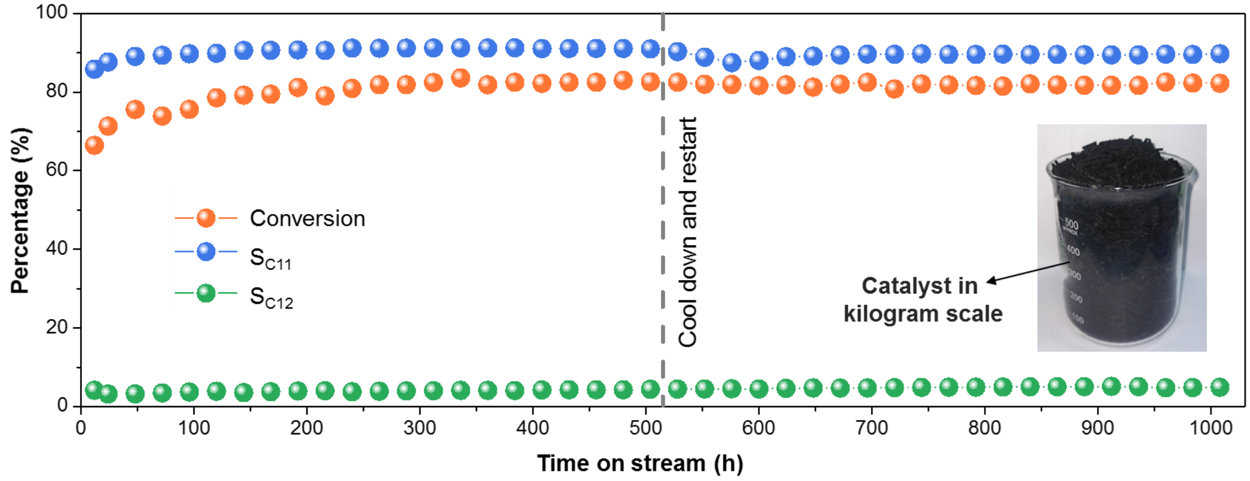


**Supplementary Fig. 32.** Catalytic performance of the bulk NiAl-P350 (synthesized on a kilogram scale and shaped into extrudates) using methyl laurate as a feedstock. Reaction conditions: T=350 ℃, $P_{H2}$=3.0 MPa, WHSV=9.4 $h^{-1}$.

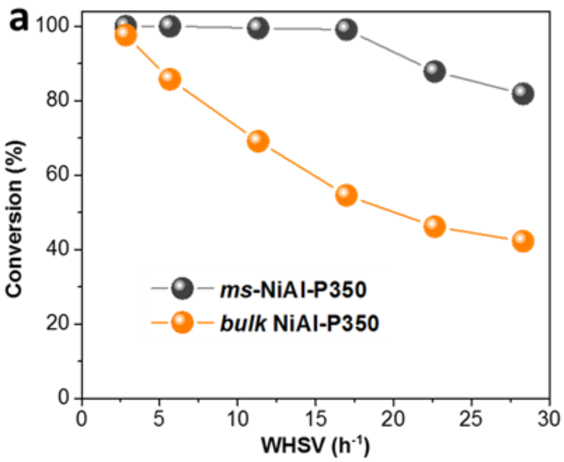


b

| Entry | WHSV ($h^{-1}$) | Con. (%) | | Reaction rate ($mmol \cdot g^{-1} \cdot h^{-1}$) | |
|---|---|---|---|---|---|
| | | *ms* | *bulk* | *ms* | *bulk* |
| 1 | 2.8 | 100 | 97.6 | 13.2 | 12.9 |
| 2 | 5.7 | 100 | 85.8 | 26.4 | 22.7 |
| 3 | 11.3 | 99.5 | 69.1 | 52.6 | 36.5 |
| 4 | 17.0 | 99.1 | 54.6 | 78.5 | 43.3 |
| 5 | 22.6 | 87.9 | 46.2 | 92.8 | 48.8 |
| 6 | 28.3 | 81.9 | 42.3 | 108.1 | 55.8 |

**Supplementary Fig. 33. (a)** Conversion of methyl laurate as a function of WHSV value over *ms*-NiAl-P350 and bulk NiAl-P350. **(b)** The calculated reaction rates over *ms*-NiAl-P350 and bulk NiAl-P350. Reaction conditions: T=350 °C, $P_{H2}$=3.0 MPa.

The catalytic reaction rates were calculated using the equation:

$$R\ (mmol \cdot g^{-1} \cdot h^{-1}) = \frac{WHSV \times X_{ML}}{214.3} \times 1000$$

Where WHSV ($h^{-1}$) is the mass flow rate of methyl laurate divided by the mass of catalyst, $X_{ML}$ (%) denotes the conversion of methyl laurate, 214.3 (g mol$^{-1}$) is the molar mass of methyl laurate.

To better compare the catalytic activities of the two catalysts, their reaction rates should be calculated and compared before a complete conversion and under a unified standard. When compared at identical conversion levels, the reaction rate is calculated to be 92.8 mmol $g^{-1}$ $h^{-1}$ for *ms*-NiAl-P350 (87.9% conversion, entry 5, Supplementary Fig. 33b), which is about four times higher than that for bulk NiAl-P350 (85.8% conversion, entry 2, Supplementary Fig. 33b). If the WHSV value is maintained constant, the reaction rate over *ms*-NiAl-P350 is about two times higher than that over bulk NiAl-P350 (entry 5 and 6, Supplementary Fig. 33b).

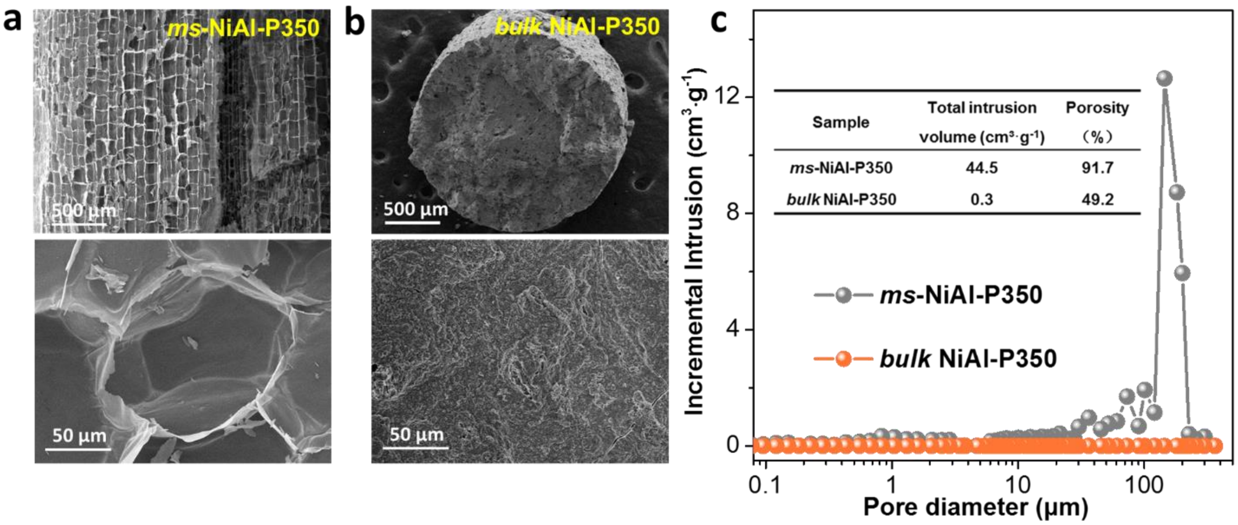


**Supplementary Fig. 34. (a)** SEM images of *ms*-NiAl-P350. **(b)** SEM images of bulk NiAl-P350. **(c)** Pore size distributions of *ms*-NiAl-P350 and bulk NiAl-P350 measured by mercury intrusion porosimetry.

The *ms*-NiAl-P350 has well-developed porous channels replicated from the *ms*-template (Supplementary Fig. 34a), but there are only a few macropores visible in the bulk NiAl-P350 (Supplementary Fig. 34b). According to the mercury intrusion porosimetry measurement (Supplementary Fig. 34c), the total intrusion volume of *ms*-NiAl-P350 is 44.5 $cm^{-3}$ $g^{-1}$ due to the presence of abundant macropores (ranging from 30-120 μm), which is much higher than that of bulk NiAl-P350 (0.3 $cm^{-3}$ $g^{-1}$).

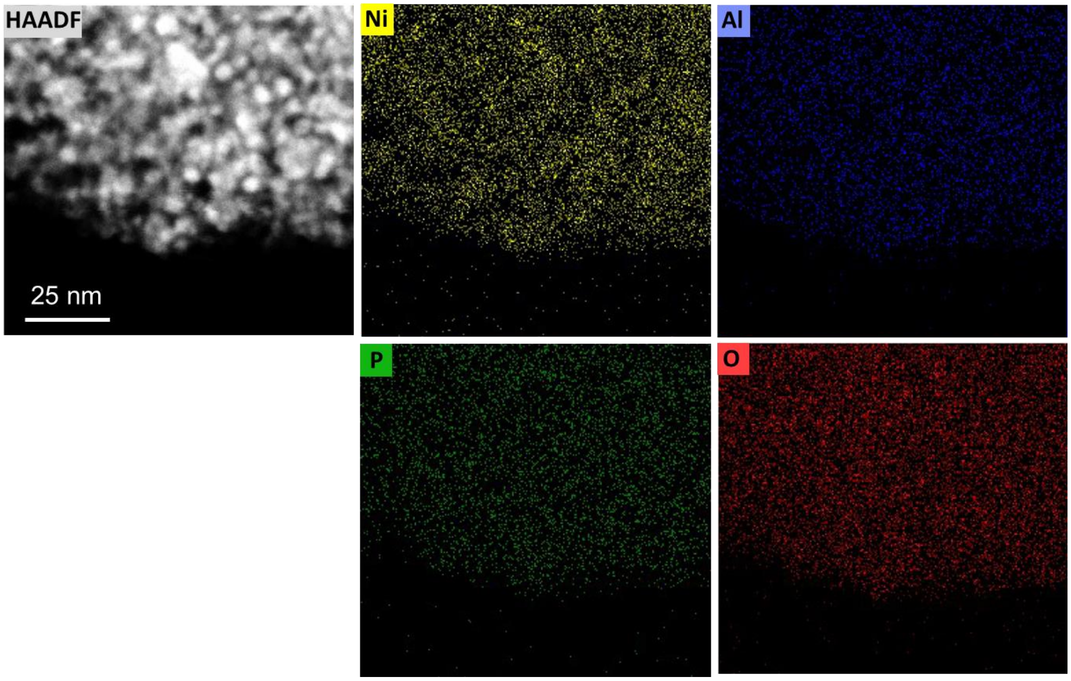


**Supplementary Fig. 35.** HAADF-STEM and the corresponding EDS elemental mapping images of bulk NiAl-P350.

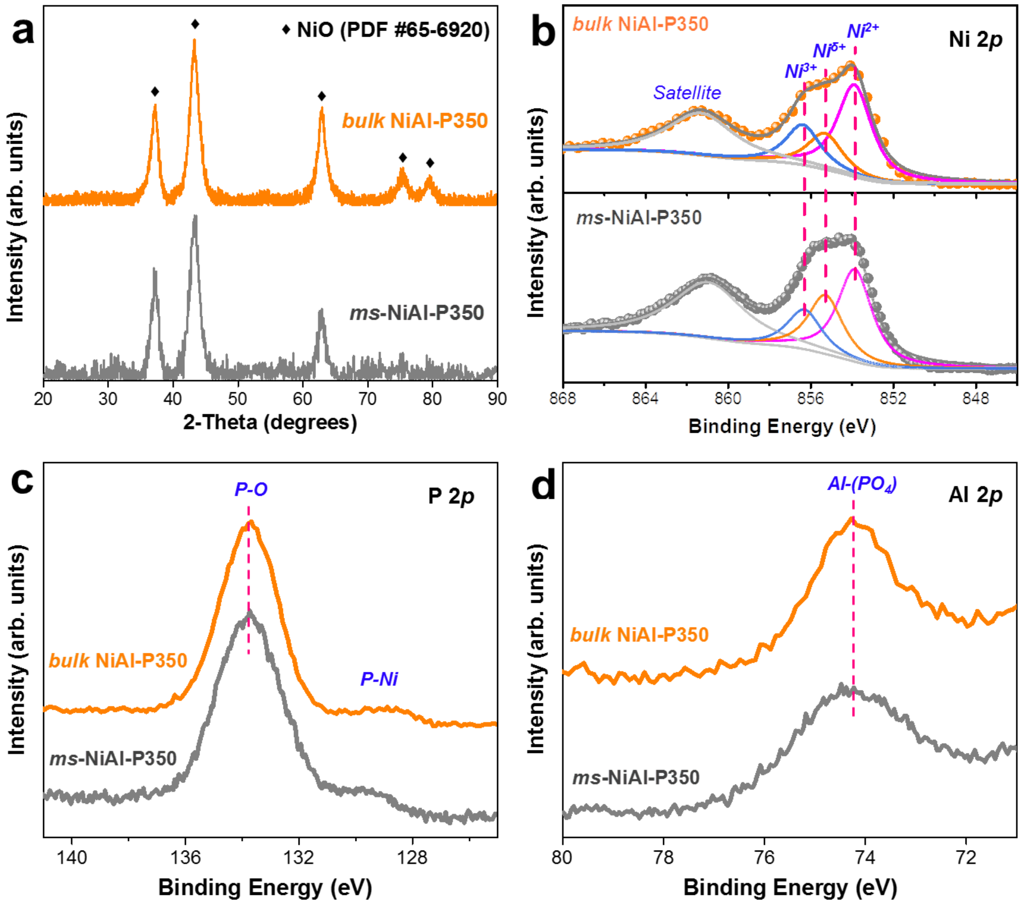


**Supplementary Fig. 36.** Comparison of structural characterization between *ms*-NiAl-P350 and bulk NiAl-P350: **(a)** XRD patterns, XPS spectra in (**b**) Ni 2$p_{3/2}$ region, (**c**) P 2*p* region and (**d**) Al 2*p* region.

**Supplementary Table 1.** Elemental contents of samples determined by ICP-OES.

| **Sample** | **wt%** | | | **P/Ni atomic ratio** |
|---|---|---|---|---|
| | **Ni** | **Al** | **P** | |
| ***ms*-NiAl** | 55.30 | 9.38 | --- | --- |
| ***ms*-NiAl-P300** | 46.39 | 8.76 | 6.69 | 0.27 |
| ***ms*-NiAl-P350** | 46.25 | 7.56 | 10.83 | 0.44 |
| ***ms*-NiAl-P450** | 36.27 | 6.29 | 23.15 | 1.21 |
| ***ms*-NiAl-P350-Sp.24h** | 48.15 | 7.83 | 7.22 | 0.28 |
| ***ms*-NiAl-P350-Sp.500h** | 48.21 | 7.85 | 6.74 | 0.26 |
| ***ms*-NiAl-P450-Sp.24h** | 51.35 | 8.52 | 24.06 | 0.89 |

**Supplementary Table 2.** Elemental contents of samples determined by XPS analysis.

| Sample | Atomic percentage (%) | | | P/Ni |
|---|---|---|---|---|
| | Ni | Al | P | |
| ***ms*-NiAl** | 22.1 | 10.2 | 0 | --- |
| ***ms*-NiAl-P300** | 17.8 | 10.1 | 7.2 | 0.41 |
| ***ms*-NiAl-P350** | 13.0 | 7.6 | 11.8 | 0.91 |
| ***ms*-NiAl-P450** | 8.4 | 3.8 | 16.5 | 1.96 |
| ***ms*-NiAl-P350-Sp.24h** | 11.5 | 8.6 | 8.2 | 0.72 |
| ***ms*-NiAl-P350-Sp.500h** | 10.6 | 10.4 | 9.1 | 0.85 |
| ***ms*-NiAl-P450-Sp.24h** | 5.6 | 7.4 | 15.4 | 2.77 |

**Supplementary Table 3.** Textural properties of samples.

| Sample | BET surface Area ($m^2g^{-1}$) | Pore Volume ($cm^3g^{-1}$) | Average pore width (nm) | Apparent density ($mg{\cdot}cm^{-3}$) |
|---|---|---|---|---|
| **NiAl powder** | 134.8 | 0.6 | 18.7 | 232.3 |
| ***ms*-NiAl** | 248.9 | 0.7 | 12.9 | 9.6 |
| **NiAl-P350 powder** | 82.4 | 0.5 | 23.8 | 307.1 |
| ***ms*-NiAl-P350** | 103.4 | 0.4 | 17.7 | 11.7 |
| ***bulk* NiAl-P350** | 78.3 | 0.3 | 14.0 | 2770.0 |

**Supplementary Table 4.** Ni 2*p* XPS data of samples.

| Sample | Position (eV) | Attribution | Percentage (%) |
|---|---|---|---|
| ***ms-NiO*** | 854.0 | $Ni^{2+}$ | 53.2 |
| | 855.9 | $Ni^{3+}$ | 46.8 |
| ***ms*-NiAl** | 854.3 | $Ni^{2+}$ | 51.3 |
| | 855.7 | $Ni^{\delta+}$ | 34.0 |
| | 856.9 | $Ni^{3+}$ | 14.7 |
| ***ms*-NiAl-P300** | 854.1 | $Ni^{2+}$ | 52.3 |
| | 855.6 | $Ni^{\delta+}$ | 36.4 |
| | 856.9 | $Ni^{3+}$ | 11.2 |
| ***ms*-NiAl-P350** | 853.8 | $Ni^{2+}$ | 48.0 |
| | 855.3 | $Ni^{\delta+}$ | 31.9 |
| | 856.4 | $Ni^{3+}$ | 20.1 |
| ***ms*-NiAl-P450** | 852.5 | Ni-P | 42.5 |
| | 855.3 | $Ni^{\delta+}$ | 13.9 |
| | 856.3 | $Ni^{3+}$ | 43.6 |

**Supplementary Table 5.** Ni K-edge EXAFS fitting results of samples.

| Sample | Path | CNs | R (Å) | $\sigma^2$ ($10^{-3}$ Å) |
|---|---|---|---|---|
| **NiO** | Ni-O | 6 | 2.07 | 7.3 |
| | Ni-Ni | 12 | 2.95 | 8.0 |
| ***ms*-NiAl** | Ni-O | 5.88 | 2.06 | 7.4 |
| | Ni-Al/Ni | 10.67 | 2.96 | 8.9 |
| ***ms*-NiAl-P350** | Ni-P/O | 5.54 | 2.05 | 7.6 |
| | Ni-Al/Ni | 10.79 | 2.96 | 8.7 |
| ***ms*-NiAl-P450** | Ni-P/O | 2.5 | 2.23 | 4.0 |
| | Ni-Ni/Al | 2.14 | 2.55 | 6.5 |

**Supplementary Table 6**. Performances of various catalysts reported in literature.

| No | Catalyst | Reactant | T / $P_{H2}$ (°C/MPa) | L/WHSV ($h^{-1}$) | Initial con. / sel. (%) | Run time (h) | Final con. / sel. (%)[d] | Deactivation reason | Ref. |
|---|---|---|---|---|---|---|---|---|---|
| **1** [a] | $Ni_2P/SiO_2$ $+CS_2$ | methyl laurate | 300 / 3 | 5 | 95 / 89 | **28** | 71/ NM | carbon deposition | 1 |
| **2** | NiZnAl | methyl laurate | 400 / 3 | 70 | NM | **3** | ~100 / 96.2 | NM | 2 |
| **3** | MoP | methyl laurate | 380 / 3 | 5 | ~100 / >90 | **102** | 84.8 / >90 | carbon deposition | 3 |
| **4** | MoRuP | | | | ~100 / 94 | **102** | 50.7 / ~94 | NM | |
| **5** | NiMoP | methyl laurate | 340 / 3 | 14 | 97.8 / ~98 | **132** | 77 / ~88 | carbon deposition | 4 |
| **6** | $Ni_2P/SiO_2$ | methyl laurate | 340 / 3 | 5 | 98 / >96 | **100** | 98/ >96 | NM | 5 |
| **7** | Ni/SAPO-11 | methyl laurate | 360 / 3 | 8 | 99 / 74.7 | **101** | 71 / 84 | sintering and carbon deposition | 6 |
| **8** | $Ni_2P$/SAPO-11 | | | | 95 / 96 | **101** | 95 / 96 | | |
| **9** | MoWC/C | canola oil | 250 / 3.1 | 5 | 100 / 78 | **3** | 60 / 45 | oxidation or leaching | 7 |
| **10** | NiP/NaMOR | palm oil | 425 / 5 | 0.9 | ~100 / 81 | **9** | ~100 / 89.5 | NM | 8 |
| **11** | $Ni_2P/SiO_2$ | methyl palmitate | 310 / 3 | NM | ~6 / NM | **8** | ~6 / NM | NM | 9 |
| **12** | Ni/γ-$Al_2O_3$ | palm oil | 300 / 5 | 1 | 92.2 / ~85 | **150** | 76.2 / ~72 | carbon deposition and sintering | 10 |
| **13** | Co/γ-$Al_2O_3$ | | | | 88.6 / ~83 | | 56.6 / ~53 | | |
| **14** | $Ni_xP_y$/SBA-15 | methyl oleate | 340 / 3 | 20.4 | ~100 / ~70 | **6** | ~100 / NM | carbon deposition | 11 |
| **15** [b] | $Ni_2P$/silica | soybean oil | 370 / 3 | 1 | 99.5/82.3 | **670** | 99.5/82.1 | NM | 12 |
| **16** | CaO-MgO-Ni/$Al_2O_3$ | palm oil | 375 / 3 | 1.2 | ~98 / ~68 | **20** | ~93 / ~60 | carbon deposition and sintering | 13 |
| **17** | NiP/AC | palm oil | 350 / 5 | 1 | ~100 / ~30 | **9** | ~100 / ~90 | NM | 14 |
| **18** | Pt/γ-$Al_2O_3$ | palm oil | 380 / 4 | 2 | NM / 77 | **80** | NM / 77 | NM | 15 |
| **19** | $NiAl_2O_4$ | palm oil | 300 / 5 | 2 | ~100 / 94.3 | **24** | NM / 94 | NM | 16 |
| **20** | Ni/γ-$Al_2O_3$ | | | | 99.5 / 80.1 | **24** | NM / ~68 | NM | |
| **21** | Pd/C | oleic acid | 300 / 4 | NM | 100 / 65 | **3** | 70 / 5 | NM | 17 |
| **22** | NiMo/C | methyl oleate | 260 / 3 | 10.3 mL $kg^{-1}$ $h^{-1}$ | ~19 / ~55 | **168** | ~16 / ~54 | carbon deposition | 18 |

| | | | | | | | | | |
|---|---|---|---|---|---|---|---|---|---|
| **23** | NiMo/$Al_2O_3$ | | | | ~23 / ~59 | **168** | ~8 / ~58 | | |
| **24** | NiMo/$SiO_2$ | | | | ~17 / ~20 | **160** | ~10 / ~25 | | |
| **25** | PtSn/SAPO-11 | palm acid | 350 / 3 | 1 | NM | **3.5** | 99.6 / 88.8 | NM | 19 |
| **26** | NiCu/γ-$Al_2O_3$ | methyl laurate | 380 / 2 | 1.5 | ~90 / ~92 | **6** | ~90 / ~90 | NM | 20 |
| **27** [c] | NiMoS/$Al_2O_3$ +$H_2S$ | methyl oleate | 260 / 6 | 6.5 | ~9 / ~43 | **600** | ~5 / ~52 | heavy products on the catalyst surface | 21 |
| **28** [c] | | oleic acid | | | ~26 / ~55 | **300** | ~45 / ~53 | | |
| **29** [c] | | triolein | | | ~38 / ~63 | **400** | ~39 / ~65 | | |
| **30** | NiMo/γ-$Al_2O_3$ | rubber seed oil | 400 / 8 | 1 | 100 / 82.2 | **18** | 100 / 75.7 | carbon deposition | 22 |
| **31** | Ni/H-Y zeolite | rubber seed oil | 350 / 3.5 | 1 | NM | **3** | 99 / 34.1 | NM | 23 |
| **32** | $CoMoS_x$ /$Al_2O_3$ | soybean oil | 350 / 5 | 2 | 80.9/NM | **6** | 47.7/NM | carbon deposition | 24 |
| **33** | $Mo_2C$/RGO | | | | 71.8 / NM | | 61.7 /NM | | |
| **34** | Ni-MoS/Zr-K | jojoba oil | 350 / 3 | 1.5 | 77 / 55 | **36** | ~92 / ~52 | NM | 25 |
| **35** | Pt/beta-$Al_2O_3$ | FAMEs | 350 / 2 | NM | >99 / NM | **160** | 98.9 / NM | NM | 26 |
| **36** | NiMoCe/$Al_2O_3$ | jatropha oil | 370 / 3.5 | 0.9 | 89 / 90 | **163** | ~80 / ~90 | NM | 27 |
| **37** | NiMoLa/$Al_2O_3$ | jatropha oil | 370 / 3.5 | 0.9 | ~86 / 94 | **177** | ~72 / ~93 | NM | 28 |
| **38** | Ni-HPW/nHA | jatropha oil | 360 / 3 | 2 | 100 / NM | **196** | 100 / NM | NM | 29 |
| **39** | NiMoC/Al-SBA-15 | soybean oil | 400 / 4.5 | 1 | 100 / 97 | **168** | 100 / 97 | NM | 30 |
| **40** | NiWC/Al-SBA-15 | corn oil | 400 / 4.5 | 1 | 100 / 100 | **384** | ~97 / ~60 | metal loss | 31 |
| **41** | *ms*-NiAl-P350 | soybean oil | 350 /0.5-3 | 6.0 | >99 />90 | **>500** | >99 />90 | no deactivation | **This work** |
| **42** | | methyl laurate | | 28.3 | 74.6 / 95.1 | **>500** | 88.5 /96.5 | | |

[a] NM means “not mentioned”.

[b] We repeated the synthesis of such catalyst and found that deactivation occurred after using 150 h under our reaction conditions (Fig. 5a).

[c] A continuous replenishment of $H_2S$ is necessary to maintain the catalyst activity.

[d] The activity retention in the main text (Fig. 3f) is calculated using the following equation:

$$\text{Activity retention (\%)} = \frac{\text{Final conversion}}{\text{Initial conversion}} \times 100\%$$

When the final conversion is larger than the initial conversion, the activity retention is set as 100%.

**Supplementary Table 7.** Fatty acid composition of various feedstocks used in this work.

| Fatty acid [a] | Fatty acid composition (wt.%) | | | |
|---|---|---|---|---|
| | **Palm oil** | **Duck fat** | **Soybean oil** | **Waste cooking oil** |
| **C14:0** | 0.99 | 0.77 | 0.12 | 0.81 |
| **C16:1** | 0.20 | 3.97 | 0.10 | 4.79 |
| **C16:0** | 37.55 | 23.67 | 10.57 | 22.60 |
| **C18:3** | 0.08 | 0.16 | 0.19 | 0.21 |
| **C18:2** | 9.37 | 17.29 | 54.35 | 15.37 |
| **C18:1** | 46.17 | 44.10 | 28.25 | 46.43 |
| **C18:0** | 4.89 | 7.80 | 4.76 | 7.46 |
| **Others** | 0.75 | 2.24 | 1.66 | 2.34 |

[a] The first number represents the number of carbon atoms, the second number is the number of double bonds in the molecule.

## References

1. Zheng, Z., Li, M.-F., Chu, Y. & Chen, J.-X. Influence of $CS_2$ on performance of $Ni_2P/SiO_2$ for deoxygenation of methyl laurate as a model compound to hydrocarbons: Simultaneous investigation on catalyst deactivation. *Fuel Process. Technol.* **134**, 259-269 (2015).
2. Pan, Z., Wang, R. & Chen, J. Deoxygenation of methyl laurate as a model compound on Ni-Zn alloy and intermetallic compound catalysts: Geometric and electronic effects of oxophilic Zn. *Appl. Catal. B: Environ.* **224**, 88-100 (2018).
3. Nie, Z., Zhang, Z. & Chen, J. Effect of Ni and noble metals (Ru, Pd and Pt) on performance of bifunctional MoP/SiO2 for hydroconversion of methyl laurate. *Appl. Surf. Sci.* **420**, 511-522 (2017).
4. Pan, Z., Wang, R., Nie, Z. & Chen, J. Effect of a second metal (Co, Fe, Mo and W) on performance of $Ni_2P/SiO_2$ for hydrodeoxygenation of methyl laurate. *J. Energy Chem.* **25**, 418-426 (2016).
5. Chen, J., Han, M., Zhao, S., Pan, Z. & Zhang, Z. An in situ approach to preparing $Ni_2P/SiO_2$ catalyst under mild conditions and its performance for the deoxygenation of methyl laurate to hydrocarbons. *Catal. Sci. Technol.* **6**, 3938-3949 (2016).
6. Zhao, S., Li, M., Chu, Y. & Chen, J. Hydroconversion of methyl laurate as a model compound to hydrocarbons on bifunctional $Ni_2P$/SAPO-11: Simultaneous comparison with the performance of Ni/SAPO-11. *Energ. Fuel.* **28**, 7122-7132 (2014).
7. Tran, C.-C., Akmach, D. & Kaliaguine, S. Hydrodeoxygenation of vegetable oils over biochar supported bimetallic carbides for producing renewable diesel under mild conditions. *Green Chem.* **22**, 6424-6436 (2020).
8. Rakmae, S. *et al.* Defining nickel phosphides supported on sodium mordenite for hydrodeoxygenation of palm oil. *Fuel Process. Technol.* **198**, 106236 (2020).
9. Han, F., Guan, Q. & Li, W. Deoxygenation of methyl palmitate over $SiO_2$-supported nickel phosphide catalysts: effects of pressure and kinetic investigation. *RSC Adv.* **5**, 107533-107539 (2015).
10. Srifa, A., Viriya-empikul, N., Assabumrungrat, S. & Faungnawakij, K. Catalytic behaviors of Ni/$\gamma$-$Al_2O_3$ and Co/$\gamma$-$Al_2O_3$ during the hydrodeoxygenation of palm oil. *Catal. Sci. Technol.* **5**, 3693-3705 (2015).
11. Yang, Y. *et al.* Influence of the Ni/P ratio and metal loading on the performance of $Ni_xP_y$/SBA-

15 catalysts for the hydrodeoxygenation of methyl oleate. *Fuel* **144**, 60-70 (2015).

12. Zarchin, R., Rabaev, M., Vidruk-Nehemya, R., Landau, M. V. & Herskowitz, M. Hydroprocessing of soybean oil on nickel-phosphide supported catalysts. *Fuel* **139**, 684-691 (2015).
13. Papageridis, K. N. *et al.* Promoting effect of CaO-MgO mixed oxide on Ni/γ-$Al_2O_3$ catalyst for selective catalytic deoxygenation of palm oil. *Renew. Energ.* **162**, 1793-1810 (2020).
14. Ruangudomsakul, M. *et al.* Influential properties of activated carbon on dispersion of nickel phosphides and catalytic performance in hydrodeoxygenation of palm oil. *Catal. Today* **367**, 153-164 (2021).
15. Jeong, H. *et al.* Superior long-term stability of a mesoporous alumina-supported Pt catalyst in the hydrodeoxygenation of palm oil. *ACS Sustain. Chem. Eng.* **9**, 1193-1202 (2021).
16. Srifa, A., Kaewmeesri, R., Fang, C., Itthibenchapong, V. & Faungnawakij, K. $NiAl_2O_4$ spinel-type catalysts for deoxygenation of palm oil to green diesel. *Chem. Eng. J.* **345**, 107-113 (2018).
17. Dragu, A. *et al.* Deoxygenation of oleic acid: Influence of the synthesis route of Pd/mesoporous carbon nanocatalysts onto their activity and selectivity. *Appl. Catal. A: Gen.* **504**, 81-91 (2015).
18. Coumans, A. E. & Hensen, E. J. M. A real support effect on the hydrodeoxygenation of methyl oleate by sulfided NiMo catalysts. *Catal. Today* **298**, 181-189 (2017).
19. Kim, K., Higai, D., Hou, X., Peng, M. & Qian, E. W. Production of normal paraffins with an even carbon number via selective hydrodeoxygenation of palm fatty acid distillate over Pt–Sn catalysts. *Ind. Eng. Chem. Res.* **60**, 2881-2889 (2021).
20. Miao, C., Zhou, G., Chen, S., Xie, H. & Zhang, X. Synergistic effects between Cu and Ni species in NiCu/γ-$Al_2O_3$ catalysts for hydrodeoxygenation of methyl laurate. *Renew. Energ.* **153**, 1439-1454 (2020).
21. Coumans, A. E. & Hensen, E. J. M. A model compound (methyl oleate, oleic acid, triolein) study of triglycerides hydrodeoxygenation over alumina-supported NiMo sulfide. *Appl. Catal. B: Environ.* **201**, 290-301 (2017).
22. Ameen, M. *et al.* Parametric studies on hydrodeoxygenation of rubber seed oil for diesel range hydrocarbon production. *Energ. Fuel.* **34**, 4603-4617 (2020).
23. Ameen, M. *et al.* H-Y zeolite as hydrodeoxygenation catalyst for diesel range hydrocarbon production from rubber seed oil. *Mater. Today: Proc.* **16**, 1742-1749 (2019).
24. Kim, S. K., Yoon, D., Lee, S.-C. & Kim, J. $Mo_2C$/graphene nanocomposite as a

hydrodeoxygenation catalyst for the production of diesel range hydrocarbons. *ACS Catal.* **5**, 3292-3303 (2015).

25. Ramesh, A., Tamizhdurai, P. & Shanthi, K. Catalytic hydrodeoxygenation of jojoba oil to the green-fuel application on Ni-MoS/Mesoporous zirconia-silica catalysts. *Renew. Energ.* **138**, 161-173 (2019).
26. Ouyang, Q. *et al.* 0.7 wt% Pt/beta-$Al_2O_3$ as a highly efficient catalyst for the hydrodeoxygenation of FAMEs to diesel-range alkanes. *Catal. Commun.* **120**, 46-50 (2019).
27. Liu, J., Fan, K., Tian, W., Liu, C. & Rong, L. Hydroprocessing of Jatropha oil over NiMoCe/$Al_2O_3$ catalyst. *Int. J. Hydrogen Energy* **37**, 17731-17737 (2012).
28. Liu, J., Liu, C., Zhou, G., Shen, S. & Rong, L. Hydrotreatment of Jatropha oil over NiMoLa/$Al_2O_3$ catalyst. *Green Chem.* **14**, 2499-2505 (2012).
29. Fan, K., Liu, J., Yang, X. & Rong, L. Hydrocracking of Jatropha oil over Ni-$H_3PW_{12}O_{40}$/nano-hydroxyapatite catalyst. *Int. J. Hydrogen Energ.* **39**, 3690-3697 (2014).
30. Wang, H., Yan, S., Salley, S. O. & Simon Ng, K. Y. Support effects on hydrotreating of soybean oil over NiMo carbide catalyst. *Fuel* **111**, 81-87 (2013).
31. Al Alwan, B., Sari, E., Salley, S. O. & Ng, K. Y. S. Effect of metal ratio and preparation method on nickel-tungsten carbide catalyst for hydrocracking of distillers dried grains with solubles corn oil. *Ind. Eng. Chem. Res.* **53**, 6923-6933 (2014).
